# Russians are the fastest 100-km ultra-marathoners in the world

**DOI:** 10.1371/journal.pone.0199701

**Published:** 2018-07-11

**Authors:** Beat Knechtle, Pantelis Theodoros Nikolaidis, Fabio Valeri

**Affiliations:** 1 Medbase St. Gallen Am Vadianplatz, St. Gallen, Switzerland; 2 Institute of Primary Care, University of Zurich, Zurich, Switzerland; 3 Exercise Physiology Laboratory, Nikaia, Greece; Universita degli Studi di Verona, ITALY

## Abstract

**Objectives:**

A recent study investigating the top 10 100-km ultra-marathoners by nationality showed that Japanese runners were the fastest worldwide. This selection to top athletes may lead to a selection bias and the aim of this study was to investigate from where the fastest 100-km ultra-marathoners originate by considering all finishers in 100-km ultra-marathons since 1959.

**Methods:**

We analysed data from 150,710 athletes who finished a 100-km ultra-marathon between 1959 and 2016. To get precise estimates and stable density plots we selected only those nationalities with 900 and more finishes resulting in 24 nationalities. Histograms and density plots were performed to study the distribution of race time. Crude mean, standard deviation, median, interquartile range (IQR), mode, skewness and excess of time for each nationality were computed. A linear regression analysis adjusted by sex, age and year was performed to study the race time between the nationalities. Histograms, density and scatter plots showed that some races seemed to have a time limit of 14 hours. From the complete dataset the finishes with more than 14 hours were removed (truncated dataset) and the same descriptive plots and analysis as for the complete dataset were performed again. In addition to the linear regression a truncated regression was performed with the truncated dataset to allow conclusion for the whole sample. To study a potential difference between races at home and races abroad, an interaction term race site home/abroad with nationality was included in the model.

**Results:**

Most of the finishes were achieved by runners from Japan, Germany, Switzerland, France, Italy and USA with more than 260’000 (85%) finishes. Runners from Russia and Hungary were the fastest and runners from Hong Kong and China were the slowest finishers.

**Conclusion:**

In contrast to existing findings investigating the top 10 by nationality, this analysis showed that ultra-marathoners from Russia, not Japan, were the fastest 100-km ultra-marathoners worldwide when considering all races held since 1959.

## Introduction

Ultra-marathon running, such as 100-km race, is a sport of increasing popularity [1]. Particularly, the number of finishers in 100-km ultra-marathon running increased exponentially, both for women and men, from 1998 to 2011 [2]. Performance in this sport depends on physiological, *e*.*g*. peak running velocity during a graded exercise test, anaerobic threshold, maximal oxygen uptake (VO_2_max) and oxygen uptake (VO_2_) at 16 km/h [3] and psychological characteristics, *e*.*g*. cognitive function [4].

Another performance-related characteristic is pacing as it has been shown that faster runners exhibit smaller decrease in their speed during a race than slower runners [5]. With regards to their training characteristics, ultra-marathoners have ~8 years of experience in ultra-running and show higher training volume and lower intensity than marathoners [1]. Training characteristics, such as weekly training distance and training pace, are predictors of 100-km ultra-marathon performance [6]. Also, anthropometric characteristics as age, body mass, body mass index and body fat correlate with performance in this sport; however, they might be less important than training characteristics [7, 8]. Furthermore, performance in 100-km ultra-marathon running is influenced by nationality and origin of the runners [2].

It is well known that athletes of a certain origin are the fastest in certain sport disciplines. For example in running, the fastest marathoners originate from East Africa, especially from Kenya and Ethiopia [9]. In longer running distances such as 100-km ultra-marathon running, most athletes at world class level originate from Japan [9]. When the top 10n athletes in 100-km ultra-marathon running between 1998 and 2011 were analysed, the 10 best race times were achieved by Japanese runners for both women and men [2].

However, these results [2] might be biased since only the top athletes were considered and not the whole number of athletes competing worldwide, and have not been verified by other studies. The knowledge of the effect of nationality on 100-km ultra-marathon performance would be of great practical importance for professionals working with ultra-marathoners.

Therefore, we analysed all 100-km ultra-marathon races held worldwide between 1959 and 2016 with the aim to identify the fastest runners by nationality. Based upon previous findings we hypothesized to confirm that female and male Japanese runners would be the fastest worldwide also when considering all finishers in 100-km ultra-marathon races held since 1959.

## Materials and methods

### Ethical approval

All procedures used in the study were approved by the Institutional Review Board of Kanton St. Gallen, Switzerland with a waiver of the requirement for informed consent of the participants given the fact that the study involved the analysis of publicly available data.

### Methods

We obtained from the website www.ultra-marathon.org/ of ‘Deutsche Ultramarathon Vereinigung’ (DUV). DUV has a large data record with race data from all ultra-marathons in http://statistik.d-u-v.org/index.php. By using the link http://statistik.d-u-v.org/ each person can access the publicly available database. We used http://statistik.d-u-v.org/geteventlist.php and inserted in ‘Year’ the term ‘all’, in ‘Distance’ the term ‘100 km’ and in ‘Country’ the term ‘All’ when using the English version of the website. By clicking on ‘Go’, all 100-km ultra-marathons held worldwide are presented. This search leads to more than 4,500 races; all race results were manually downloaded by one of the investigators.

The original dataset contains the following variables: *name*, *age* at race, *year* of race, *sex* of finisher, *nationality* and *country*, and *speed* in km per hours. We converted running speed to *time* in hours by dividing 100 km by speed (km/h). To identify unique finisher we computed date of birth with year of race minus age at race. After cleaning the variable name we identified unique finisher if finisher has the same name, date of birth, sex and nationality. Finishes with missing in age, year, sex and times were removed. Finishes out of the following ranges were removed: date of birth between 1890 and 2000, age between 15 and 100, year between 1950 and 2016, and number if character in nationality and country is 3. After removing the finishes from missing data and outliers we selected only finishes which nationality has equal or more than 900 finishes. This dataset has 24 nationalities and we named it *complete dataset*. To study the distribution of time we produced histograms and density plots with Gaussian kernel for each of the selected nationality. Also we produced normal distribution for each nationality defined by the crude mean and standard deviation of each nationality to compare with the empirical distribution. Furthermore we computed crude mean, standard deviation, median, interquartile range (IQR), mode, skewness and excess kurtosis of time for each nationality. Excess kurtosis or shortened excess is defined as kurtosis minus 3. An excess of 0 means a Gaussian-like kurtosis (mesokurtic), a positive excess has a slender form of curve (leptokurtic), and negative excess has a broader curve (platykurtic).

Due to various kind of distribution of time we decided to cluster the nationalities according to time density. The range of time (h) was segmented in 0 to 7, 7 to 8, 8 to 9, …,22 to 23, 23 to 24 and ≥ 24. For each of these 19 segments we computed the area under der density curve. With that, we performed an agglomerative hierarchical clustering using the group average clustering to analyse groups of similar distribution of time.

To study the time between the nationalities we performed a linear regression analysis adjusting by sex, age and year:

$$time=sex\times (year+year^{2})+sex\times (age+age^{2})+sex\times nationality$$ (1)

**Fig 1:**
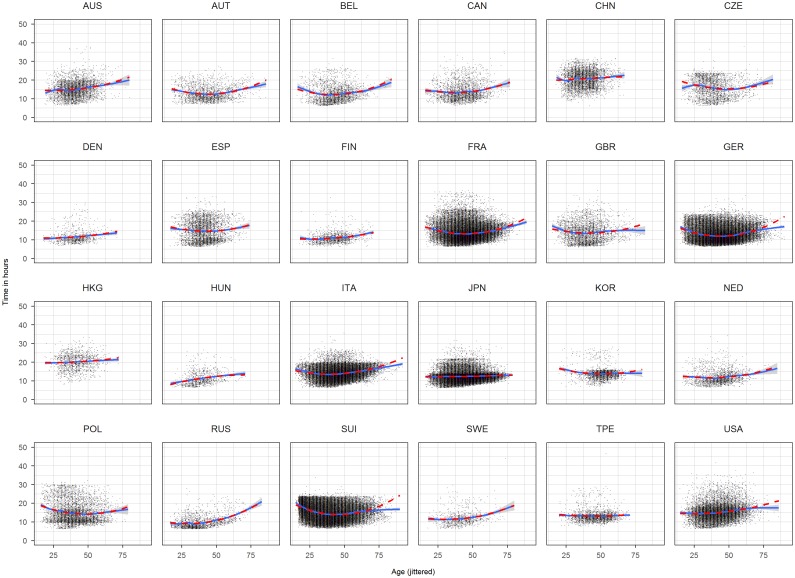
Scatterplots time against race year for each nationality based on the complete dataset. Year has been jittered.

**Fig 2:**
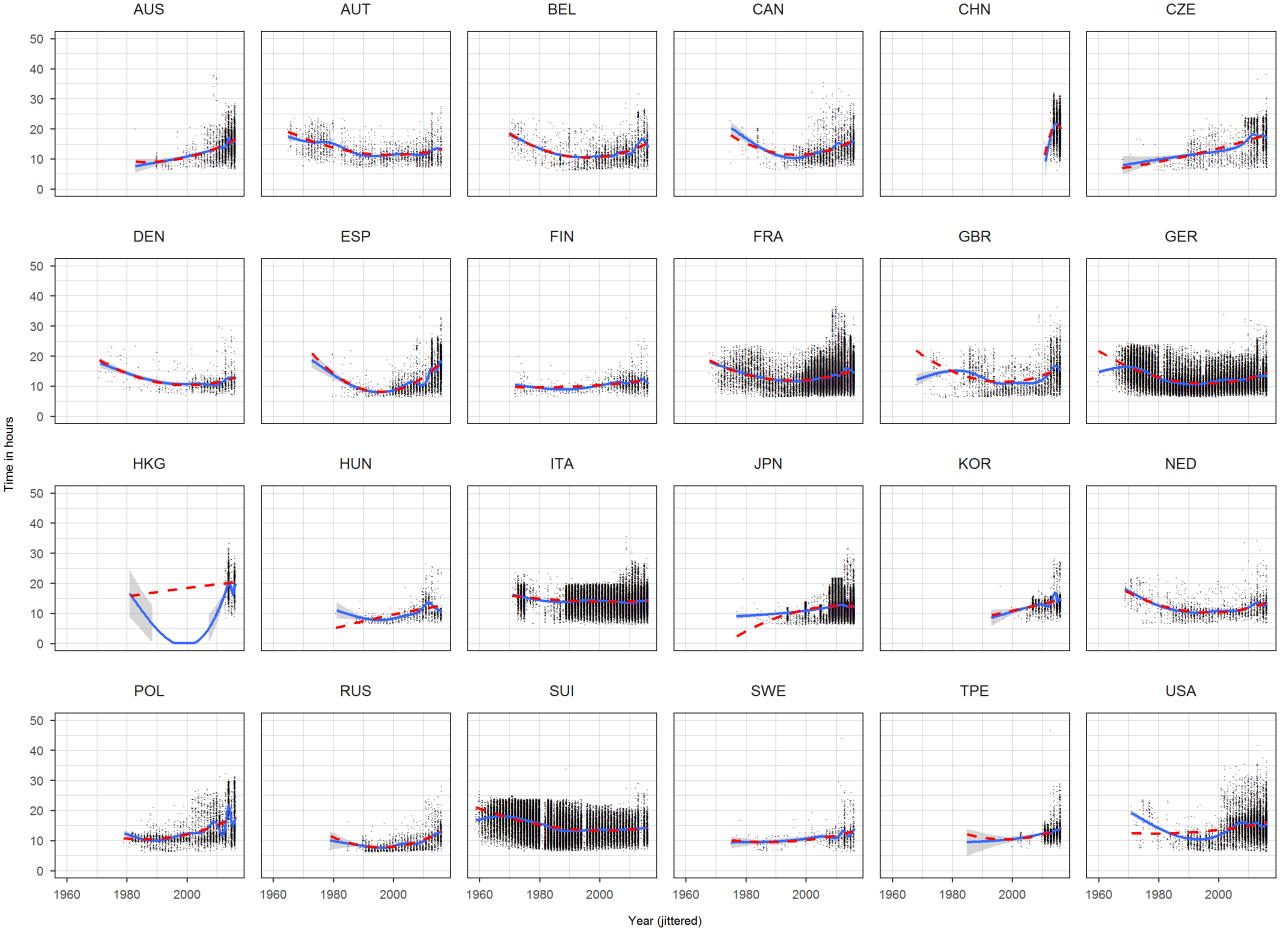
Scatterplots time against race age for each nationality based on the complete dataset. Age has been jittered.

We included a quadratic term for age and year and also an interaction term between sex and age and age squared, sex and year and year squared and sex and nationality. This model (1) is based on visual inspection of scatterplots of time against year and time against age for each nationality (Figs 1 and 2). We included the following fitting curve to each panel: a b-spline (solid) of age, respectively, time, and a quadratic term (dashed). Since both curves overlaps for the large range using a quadratic term seems admissible. The variable age and year were centered by the median with median year of 2009 and median age of 44. Reference level of sex was male and reference level of nationality was Australia (AUS).

**Fig 3:**
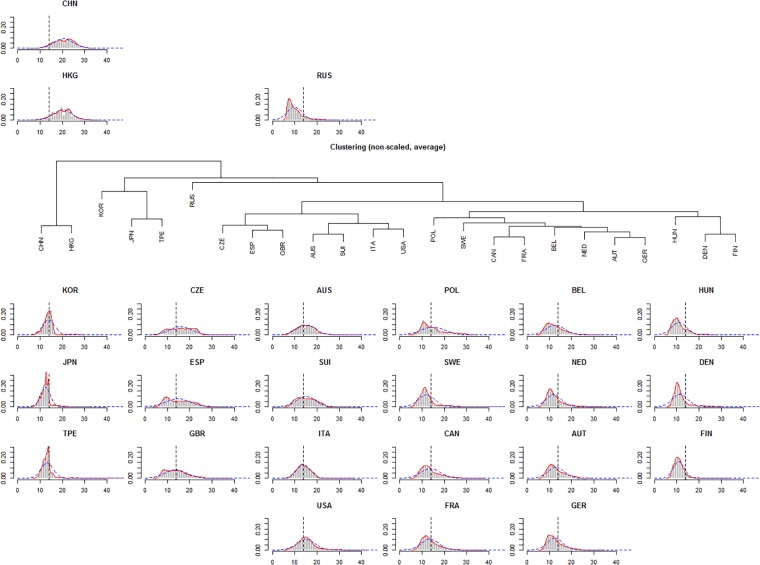
Histograms, density plots and normal distributions based on mean and standard deviation for each country. The diagrams are positioned according the hierarchical cluster analysis. Graphs are based on the complete dataset.

**Fig 4:**
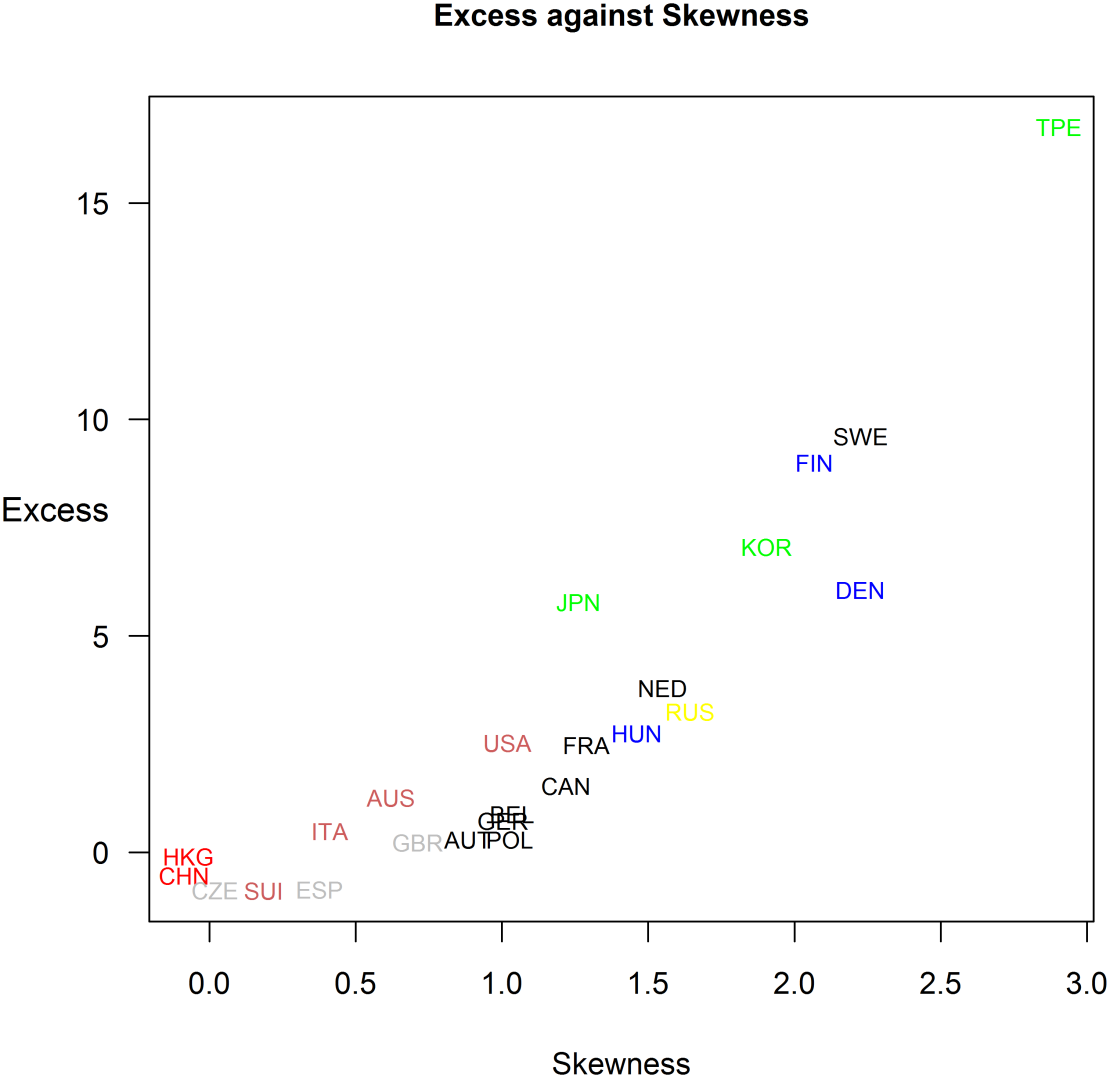
Scatterplot with excess against skewness. Groups of nation are distinguished by different colours.

**Fig 5:**
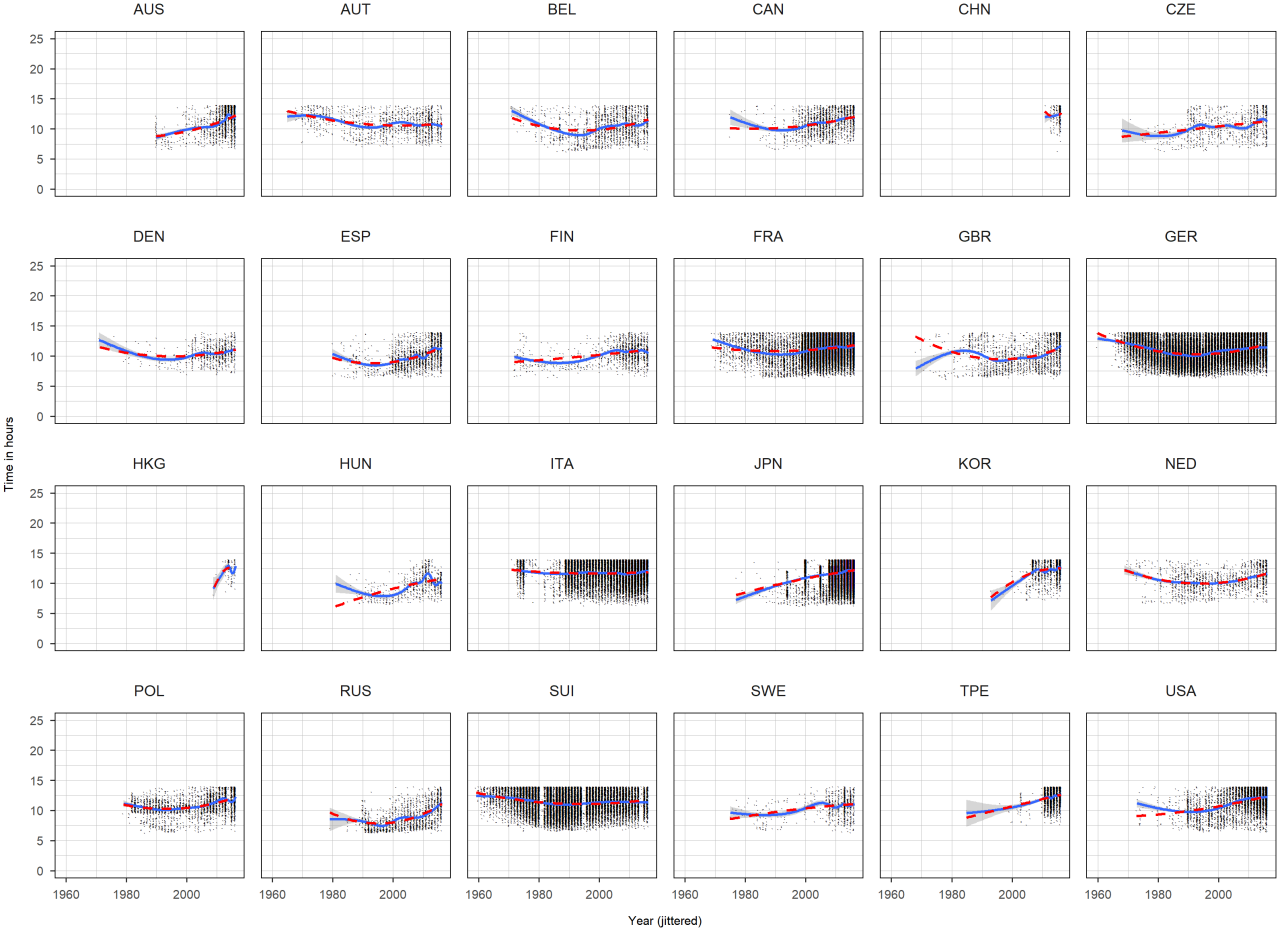
Scatterplots time against race year for each nationality based on the truncated dataset. Year has been jittered.

**Fig 6:**
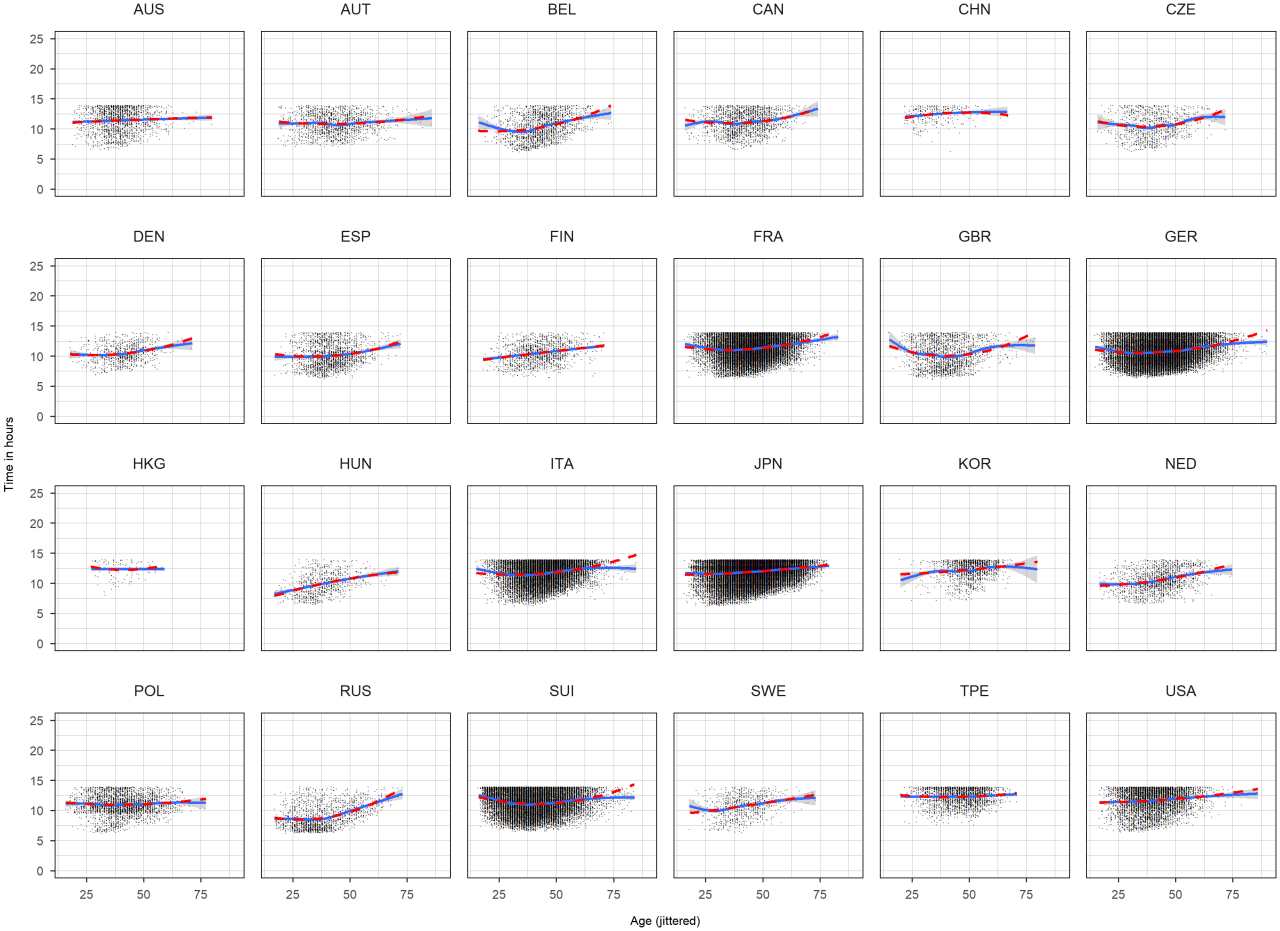
Scatterplots time against race age for each nationality based on the truncated dataset. Age has been jittered.

Histograms, density plots (Fig 3) and scatterplots (Figs 1 and 2) show that some races seem to have a time limit: finishers who didn’t reach certain time limit were discarded. To account for this limit we defined a time limit of 14 hours based on the plots and histograms of Japan, Korea and Taiwan. From the complete dataset we removed the finishes which have more than 14 hours (Fig 4) which we called the *truncated dataset* and produced the same descriptive plots and analysis as for the complete dataset (Figs 5 and 6). Additionally to the linear regression we performed a truncated regression with the truncated dataset to allow conclusion to the whole sample. The estimates from the regressions were used to compute the times of a reference finisher: median of age, median of year and male. These times and resulting ranks were compared between the various nationalities.

Furthermore, to study if there is a difference between races at home and races on abroad we included in model (1) an interaction term race site (home/abroad) with nationality and sex (2):

$$time=sex\times (year+year^{2})+sex\times (age+age^{2})+sex\times nationality\times site$$ (2)

Since 64.6% of the finisher had only one race and 16.6% had two races we performed regression analysis without including the cluster effect of finishers.

All data processing and analysis were performed with the statistical software R [10]. Truncated regression was performed with function truncreg from package truncreg.

## Results

Between 1959 and 2016, a total of 363,924 athletes finished a 100-km ultra-marathon. The variable with the highest number of missing data is date of birth. There was no missing in variable sex. Table 1 summarizes the exclusion criteria. Only nationalities with at least 900 finishes were considered to allow precise estimates and robust histogram. To analyse which country have the most missing data in variable date of birth nationalities with at least 1,000 finishes and at least 10% of missing data are listed in Table 2 by descending order of missing data. Malaysia, Korea, Portugal and Great Britain have the most missing data with 70.1%, 60.6%, 48.1%, 41.5%, respectively. Finally, a total of 150,710 finishers originating from 24 countries with a total of 307,871 finishes could be considered for data analysis.

**Table 1:** Total number, missing and out of range.

Criteria	N Finishes	N Finisher	N Nationality	% Finishes	% Finisher	% Nationality
[1] Total	363,924	195,983	128	100	100	100
[2] Exclude missing/incorrect hours	363,923	195,982	128	100	100	100
[3] Exclude missing age/date of birth	318,231	157,190	125	87.4	80.2	97.7
[4] Exclude unclear nationality	318,228	157,187	124	87.4	80.2	96.9
[5] Exclude nation < 900 finishes	307,871	150,710	24	84.6	76.9	18.8

**Table 2:** Missing data in date of birth and/or age according to nationality. Only nationalities with at least 10% missing are shown.

Nationality	N	Missing	Missing (%)
MAS	1,507	1,057	70.1
KOR	6,539	3,963	60.6
POR	1,442	693	48.1
GBR	5,834	2,419	41.5
NZL	1,119	452	40.4
FIN	2,359	917	38.9
HKG	2,123	791	37.3
CHN	6,165	1,942	31.5
ESP	5,913	1,854	31.4
TPE	3,843	1,065	27.7
JPN	79,011	17,021	21.5
DEN	1,224	263	21.5
AUS	5,103	1,093	21.4
CAN	3,093	417	13.5
NED	2,261	234	10.3
BEL	2,896	294	10.2

Table 3 presents the number of finishes by origin of the athletes. Most of the finishes were achieved by runners from Japan, Germany, Switzerland, France, Italy and USA with more than 260’000 (85%) finishes.

**Table 3:** Quantity structure of selected nationalities.

Nationality	N Finishes	N Finisher	Race at home (%)	Finishes per finisher
JPN	61'990	41'081	98.8%	1.51
GER	51'313	18'085	39.7%	2.84
SUI	49'596	17'609	98.8%	2.82
FRA	46'553	22'768	89.6%	2.04
ITA	38'177	14'766	96.2%	2.59
USA	14'356	9'627	91.6%	1.49
POL	5'472	3'112	79.4%	1.76
CHN	4'223	3'069	75.2%	1.38
ESP	4'059	2'785	78.8%	1.46
AUS	4'010	2'437	92.0%	1.65
GBR	3'414	2'301	38.7%	1.48
TPE	2'778	1'955	88.4%	1.42
CAN	2'676	1'401	69.6%	1.91
BEL	2'602	1'163	38.5%	2.24
KOR	2'576	1'486	95.9%	1.73
CZE	2'506	1'289	66.6%	1.94
AUT	2'082	969	19.5%	2.15
NED	2'027	805	62.0%	2.52
RUS	1'852	920	62.6%	2.01
FIN	1'442	479	86.8%	3.01
HKG	1'332	1'070	89.9%	1.24
DEN	960	544	70.6%	1.76
HUN	947	419	65.6%	2.26
SWE	928	570	73.0%	1.63

**Fig 7:**
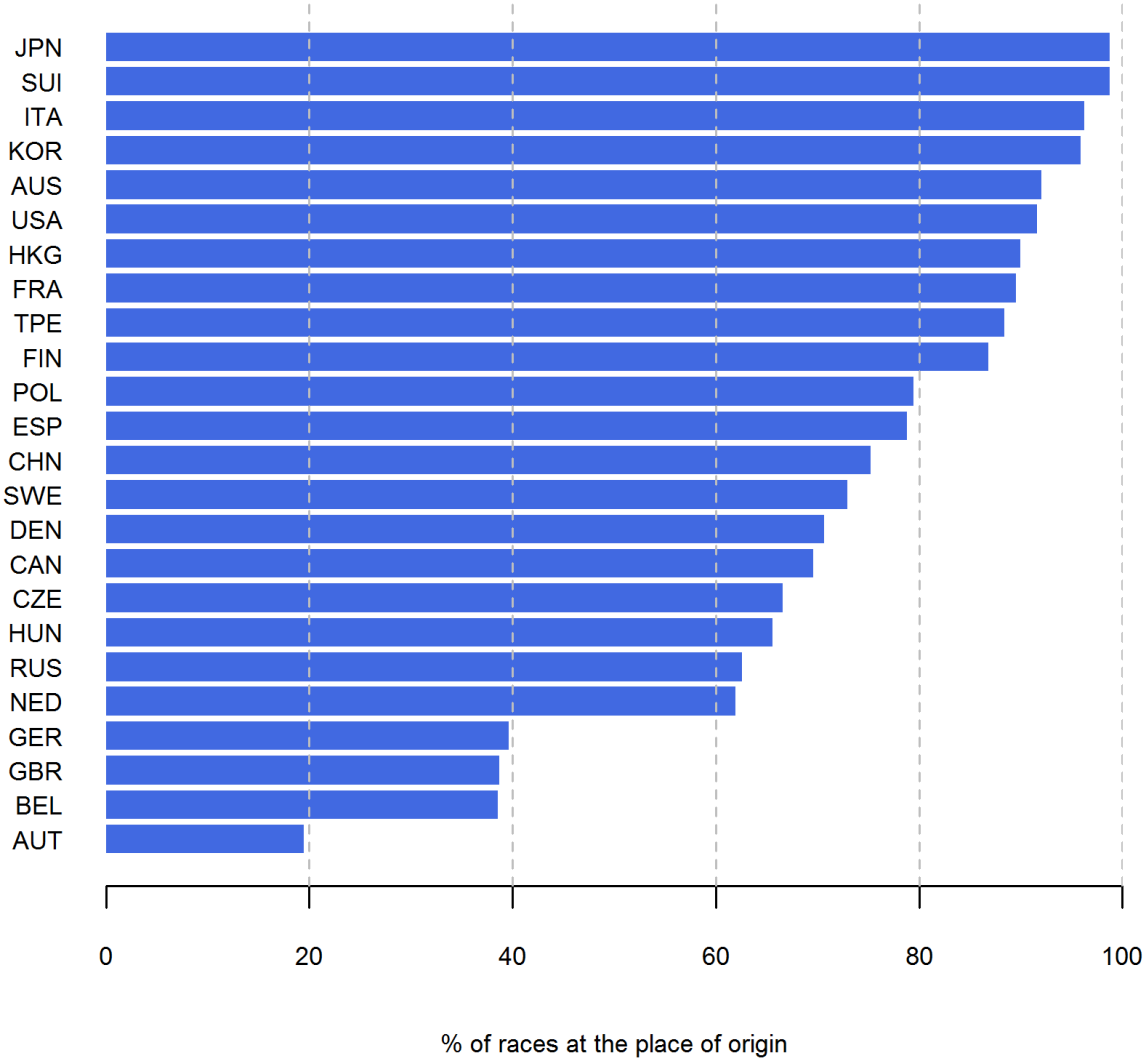
Percentage of races which takes place at the origin of the finisher.

**Fig 8:**
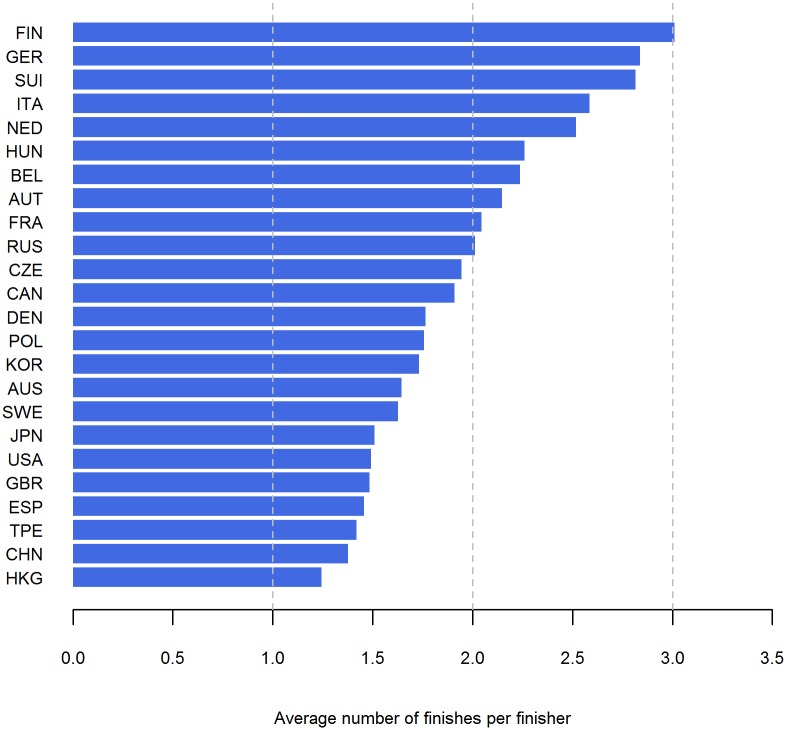
Average number of finishes. This figure is based on the complete dataset.

A total of 20 nationalities performed more than 50% of their races at their home country with runners from Japan, Switzerland, Italy and Korea on the top whereas runners from Germany, Great Britain, Belgium and Austria have performed less than 50% of the races abroad (Fig 7). Runners from Finland, Germany, Switzerland, Italy, Netherlands Hungary, Belgium, Austria, France and Russia have an average number of finishes per finisher of more than 2 (Fig 8).

A total of 64.6% of the finishers completed only one 100-km ultra-marathon (Table 4). On average, the athletes were 43.7±11.1 years old (Table 5). A total of 88% of the finishers are men and 12% are women (Table 6).

**Table 4:** Distribution of number of finishes per finisher.

N Finishes	N Finisher	Frequency in %
1	97,340	64.6
2	25,032	16.6
3	10,588	7
4	5,561	3.7
5	3,558	2.4
5–9	5,365	3.6
10–19	2,708	1.8
20–39	498	0.3
40–59	51	0
60–79	4	0
80–99	1	0
100–149	2	0

**Table 5:** Baseline of continuous variables.

Variable	Mean	SD	Median	IQR	Min	Max
Age	43.7	11.1	44	36–51	15	92
Date of birth	1959	15.3	1960	1949–1970	1891	2000
Year	2003	14	2009	1993–2014	1959	2016
Time	13.7	3.82	12.9	11–15.7	6.17	46.8

**Table 6:** Distribution of finishing according to categorical variables.

Variable	Level	N	Percent (%)
sex	Male	271,224	88
	Female	36,647	12
nat	JPN	61,990	20
	GER	51,313	17
	SUI	49,596	16
	FRA	46,553	15
	ITA	38,177	12
	USA	14,356	4.7
	POL	5,472	1.8
	CHN	4,223	1.4
	ESP	4,059	1.3
	AUS	4,010	1.3
	GBR	3,414	1.1
	TPE	2,778	0.9
	CAN	2,676	0.87
	BEL	2,602	0.85
	KOR	2,576	0.84
	CZE	2,506	0.81
	AUT	2,082	0.68
	NED	2,027	0.66
	RUS	1,852	0.6
	FIN	1,442	0.47
	HKG	1,332	0.43
	DEN	960	0.31
	HUN	947	0.31
	SWE	928	0.3
country	SUI	84,856	28
	JPN	61,702	20
	FRA	43,751	14
	ITA	40,736	13
	GER	21,943	7.1
	USA	13,770	4.5
	POL	4,404	1.4
	AUS	4,223	1.4
	ESP	3,922	1.3
	NED	3,342	1.1
	other (49)	28,564	9.3

From the agglomerative hierarchical clustering 5 groups can be retrieved:

- Group 1 with China and Hong Kong which show a wide spread distribution of time.
- Group 2 with Russia which has the lowest mode and high excess (3.2).
- Group 3 with Korea, Japan and Taiwan which have a very high excess (7.1, 5.8 and 16.8). All have a very step right curve at 18, 14 and 14 hours, respectively.
- Group 4 with Czech Republic, Spain, Great Britain, Australia, Switzerland, Italy and USA with low excess and low skewness.
- Group 5 with Finland, Denmark, Nederland, Belgium, Hungary, Poland, Sweden, France, Canada, Austria and Germany which have a higher skewness (≥1) compared to group 4 with skewness ≤ 1 (Fig 4).

Table 7 compares the number of finishes before and after truncation of the data set for each nationality. Hong Kong and China have more than 90% truncated observation, Australia, Czech Republic, Spain, Switzerland and USA have between 50% and 63% truncated observation and all others less than 50%. Table 8 presents the mean, SD, median, interquartiles, mode, skewness and excess of time for each nationality of the complete dataset and Table 9 for the truncated data set.

**Table 7:** Numbers of finishes before and after truncation and percentage of removed finishes.

Nationality	N finishes before truncation	N finishes after truncation	Removed (%)
AUS	4,010	1,702	57.6
AUT	2,082	1,417	31.9
BEL	2,602	1,810	30.4
CAN	2,676	1,728	35.4
CHN	4,223	287	93.2
CZE	2,506	940	62.5
DEN	960	826	14
ESP	4,059	1,965	51.6
FIN	1,442	1,324	8.2
FRA	46,553	29,815	36
GBR	3,414	1,871	45.2
GER	51,313	37,412	27.1
HKG	1,332	109	91.8
HUN	947	774	18.3
ITA	38,177	20,021	47.6
JPN	61,990	56,777	8.4
KOR	2,576	1,483	42.4
NED	2,027	1,612	20.5
POL	5,472	2,970	45.7
RUS	1,852	1,648	11
SUI	49,596	21,981	55.7
SWE	928	774	16.6
TPE	2,778	2,286	17.7
USA	14,356	5,819	59.5

**Table 8:** Mean, SD, median, interquartiles, mode, skewness and excess of time for each nationality of the complete dataset.

Nationality	Number of finishes	Mean (SD)	Median (IQ)	Minimum	Maximum	Mode	Skewness	Excess
AUS	4,010	15.2 (4.17)	15 (12.2–17.8)	6.62	37.9	13.6	0.62	1.256
AUT	2,082	13 (3.69)	12.2 (10.3–15.2)	7.11	27.6	10.8	0.884	0.29
BEL	2,602	12.7 (4.21)	11.8 (9.48–14.8)	6.26	31.7	9.58	1.035	0.88
CAN	2,676	13.8 (4.39)	12.7 (10.7–16)	6.68	35.6	11.6	1.22	1.535
CHN	4,223	20.7 (4.4)	20.9 (17.4–23.9)	6.31	32.3	22.9	-0.086	-0.545
CZE	2,506	16 (5)	16 (11.7–20.3)	6.3	38.2	15.7	0.02	-0.879
DEN	960	11.6 (3.36)	10.7 (9.69–12.3)	6.96	29.8	10.2	2.225	6.055
ESP	4,059	14.8 (5.3)	14.3 (9.96–19.1)	6.33	33.1	9.42	0.377	-0.864
FIN	1,442	11 (2.52)	10.7 (9.49–12.1)	6.51	32.8	10.1	2.068	8.999
FRA	46,553	13.6 (3.88)	12.8 (10.9–15.4)	6.39	36.6	11.7	1.288	2.475
GBR	3,414	13.9 (4.92)	13.4 (9.8–16.8)	6.17	36.4	8.56	0.713	0.222
GER	51,313	12.5 (3.47)	11.7 (9.9–14.4)	6.41	33.9	9.78	1.004	0.728
HKG	1,332	20.1 (4.14)	20 (17.4–23)	8.09	33.4	22.6	-0.072	-0.103
HUN	947	11.2 (3.36)	10.4 (8.82–12.7)	6.53	26.6	9.91	1.46	2.744
ITA	38,177	14 (3.04)	13.8 (11.9–16.1)	6.31	35.7	13.4	0.412	0.489
JPN	61,990	12.4 (2.13)	12.5 (11.2–13.4)	6.23	31.7	12.8	1.262	5.774
KOR	2,576	13.8 (2.76)	13.7 (12.4–14.7)	7.2	28.2	14.5	1.905	7.052
NED	2,027	12 (3.43)	11.2 (9.72–13.4)	6.64	34.2	10.2	1.55	3.787
POL	5,472	14.9 (5.41)	13.4 (10.8–17.3)	6.3	32.4	10.8	1.028	0.292
RUS	1,852	9.92 (3.23)	8.94 (7.56–11.3)	6.31	28.1	7.47	1.644	3.245
SUI	49,596	15.1 (4.03)	14.8 (11.8–18.2)	6.63	33.8	11.8	0.187	-0.89
SWE	928	12 (3.61)	11.4 (9.85–13)	6.38	44.2	11.2	2.226	9.605
TPE	2,778	13.1 (2.7)	12.9 (11.7–13.8)	7.62	46.8	13.6	2.903	16.754
USA	14,356	15.1 (3.96)	14.8 (12.6–17)	6.46	41.8	14.7	1.019	2.522

**Table 9:** Mean, SD, median, interquartiles, mode, skewness and excess of time for each nationality of the truncated dataset.

Nationality	N finishes	Mean (SD)	Median (IQ)	Minimum	Maximum	Mode	Skewness	Excess
AUS	1,702	11.4 (1.87)	11.7 (9.93–13)	6.62	14	13.4	-0.492	-0.782
AUT	1,417	10.9 (1.68)	10.9 (9.62–12.2)	7.11	14	10.5	-0.094	-0.896
BEL	1,810	10.4 (1.98)	10.3 (8.79–12.1)	6.26	14	9.58	0.015	-1.025
CAN	1,728	11.1 (1.71)	11.3 (9.84–12.6)	6.68	14	11.6	-0.249	-0.848
CHN	287	12.4 (1.29)	12.8 (11.6–13.4)	6.31	14	13.4	-1.152	1.482
CZE	940	10.6 (2)	10.6 (9.01–12.3)	6.3	14	9.56	-0.054	-1.053
DEN	826	10.5 (1.46)	10.4 (9.53–11.4)	6.96	14	10.1	0.119	-0.473
ESP	1,965	10.1 (1.88)	9.88 (8.66–11.5)	6.33	14	9.59	0.296	-0.801
FIN	1,324	10.5 (1.63)	10.5 (9.38–11.7)	6.51	14	10	0.009	-0.692
FRA	29,815	11.3 (1.64)	11.4 (10.1–12.6)	6.39	14	11.7	-0.352	-0.581
GBR	1,871	10.3 (2.14)	10.2 (8.41–12.1)	6.17	14	8.34	0.086	-1.231
GER	37,412	10.8 (1.69)	10.8 (9.48–12.1)	6.41	14	9.74	-0.027	-0.869
HKG	109	12.4 (1.39)	12.6 (11.4–13.6)	8.09	14	13.4	-0.882	0.019
HUN	774	9.91 (1.79)	9.84 (8.54–11.1)	6.53	14	10.1	0.244	-0.675
ITA	20,021	11.7 (1.6)	12 (10.6–13)	6.31	14	13.5	-0.643	-0.32
JPN	56,777	11.9 (1.5)	12.3 (11–13)	6.23	14	12.8	-0.846	0.155
KOR	1,483	12.3 (1.43)	12.7 (11.5–13.4)	7.2	14	13.5	-1.098	0.72
NED	1,612	10.6 (1.72)	10.5 (9.42–11.8)	6.64	14	10	-0.023	-0.696
POL	2,970	11 (1.64)	10.9 (10–12.2)	6.3	14	10.5	-0.312	-0.127
RUS	1,648	9.04 (1.93)	8.53 (7.39–10.5)	6.31	14	7.23	0.623	-0.647
SUI	21,981	11.3 (1.71)	11.5 (9.95–12.7)	6.63	14	11.7	-0.323	-0.814
SWE	774	10.7 (1.76)	10.9 (9.53–12)	6.38	14	11.3	-0.31	-0.627
TPE	2,286	12.3 (1.31)	12.6 (11.4–13.4)	7.62	14	13.6	-0.74	-0.135
USA	5,819	11.7 (1.79)	12 (10.5–13.1)	6.46	14	13.5	-0.776	-0.245

**Fig 9:**
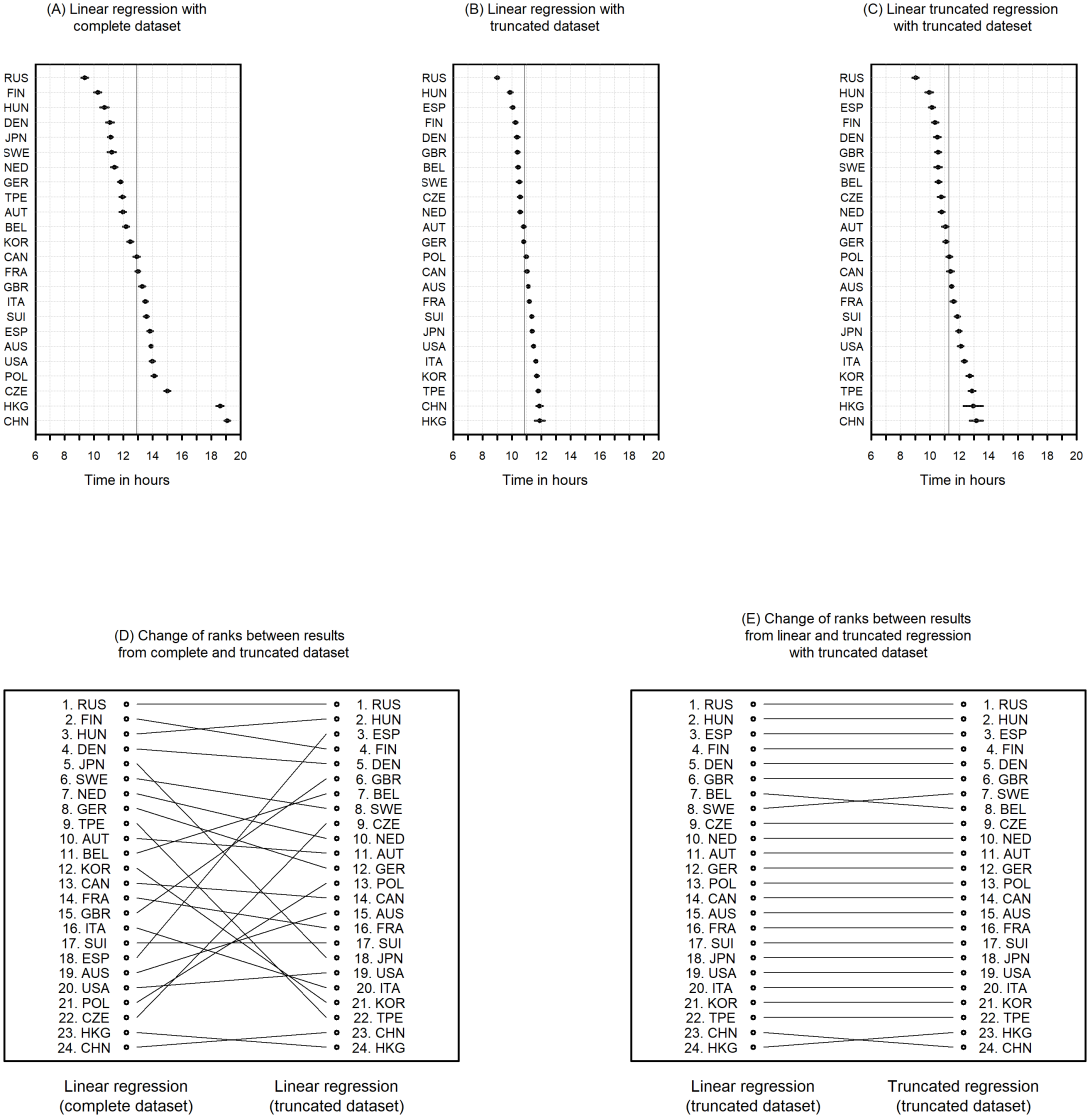
The upper panel shows the adjusted time for each nationality in ascending order at reference sex = male, age = 44 and year = 2009. (A) is based on linear regression of the complete dataset, (B) on the truncated dataset and (C) on the truncated regression of the truncated dataset. The lower panel with figures (D) and (E) shows the changes in rank from (A) to (B) and (B) to (C).

**Fig 10:**
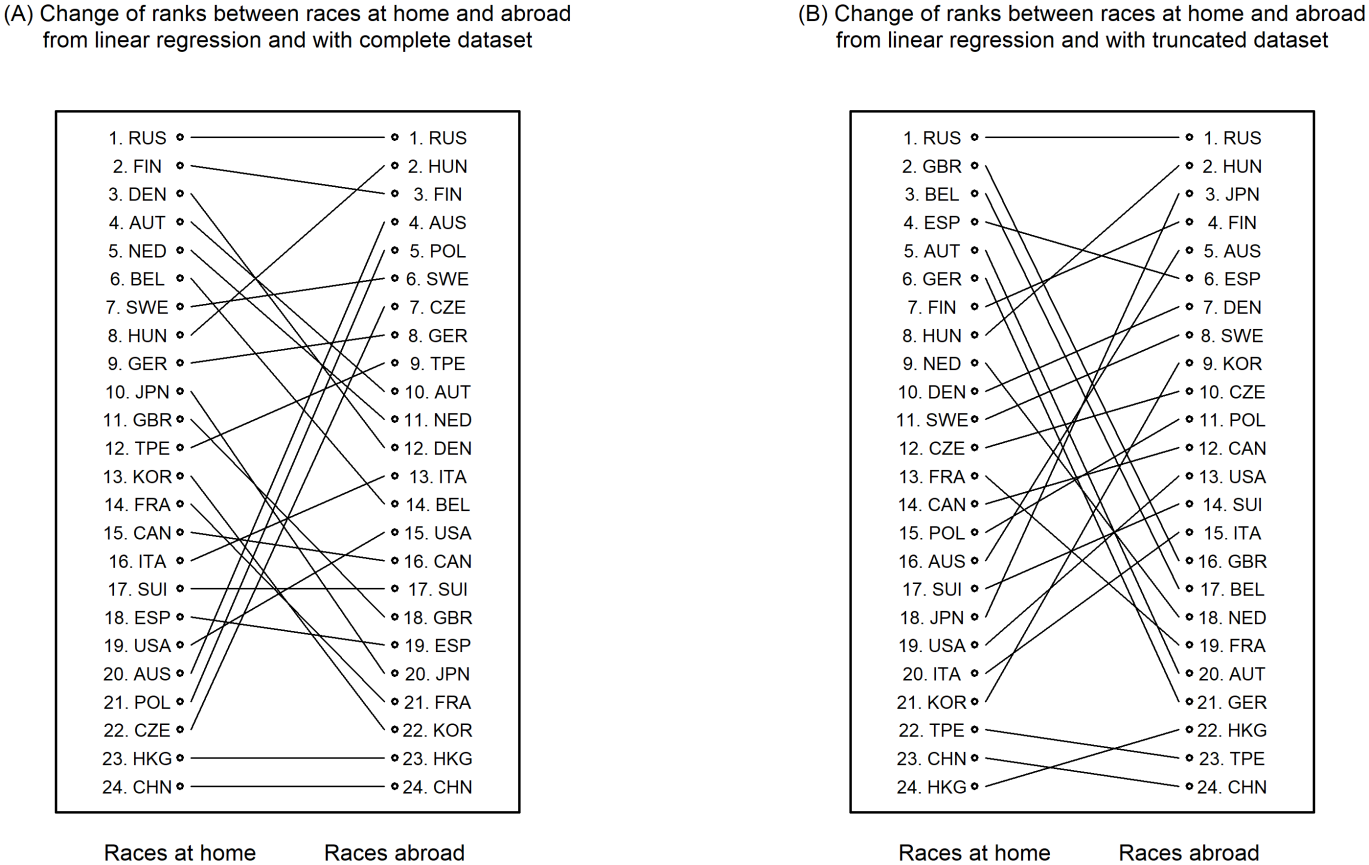
The rank of nationality computed from model 2 (interaction of nationality with races at home/abroad). (A) shows rank changes from races at home to races abroad based on linear regression with complete dataset. (B) shows rank changes from races at home to races abroad based on linear regression with truncated dataset.

Estimates, standard errors and p-values from models (1) and (2) based on complete and truncated dataset are given in Tables 10–14. These data were used to compute times at the reference sex = male, year of race = 2009 and age = 44 which are presented in Tables 15 and 16 and Figs 9 and 10.

**Table 10:** Results from linear regression with complete dataset time = sex×(year+year^2^)+sex×(age+age^2^)+sex×nationality and referenced to male, age 44, year 2009 and nationality Australia.

	Coefficient	Standard error	P-Value
Intercept	13.879	0.0600	0.000
Sex (female)	0.892	0.1254	0.000
Age	0.012	0.0006	0.000
Age squared	0.0033	0.0000	0.000
Year	0.156	0.0013	0.000
Year squared	0.0062	0.0000	0.000
Female×Age	0.022	0.0019	0.000
Female×Age squared	-0.0006	0.0001	0.000
Female×Year	0.014	0.0038	0.000
Female×Year squared	0.0016	0.0001	0.000
AUT	-1.922	0.0976	0.000
BEL	-1.713	0.0905	0.000
CAN	-0.976	0.0967	0.000
CHN	5.199	0.0808	0.000
CZE	1.104	0.0927	0.000
DEN	-2.806	0.1301	0.000
ESP	-0.069	0.0801	0.389
FIN	-3.634	0.1126	0.000
FRA	-0.898	0.0621	0.000
GBR	-0.597	0.0867	0.000
GER	-2.075	0.0634	0.000
HKG	4.708	0.1152	0.000
HUN	-3.176	0.1334	0.000
ITA	-0.378	0.0629	0.000
JPN	-2.764	0.0613	0.000
KOR	-1.425	0.0895	0.000
NED	-2.503	0.0985	0.000
POL	0.220	0.0758	0.004
RUS	-4.524	0.1064	0.000
SUI	-0.320	0.0642	0.000
SWE	-2.678	0.1339	0.000
TPE	-1.956	0.0881	0.000
USA	0.079	0.0676	0.244
AUT×Female	-0.705	0.2708	0.009
BEL×Female	-0.245	0.2660	0.357
CAN×Female	0.330	0.1868	0.077
CHN×Female	-0.070	0.1939	0.718
CZE×Female	0.629	0.2280	0.006
DEN×Female	-0.638	0.3222	0.048
ESP×Female	1.444	0.2405	0.000
FIN×Female	0.149	0.2622	0.569
FRA×Female	-0.038	0.1341	0.778
GBR×Female	-1.098	0.1905	0.000
GER×Female	-0.441	0.1372	0.001
HKG×Female	0.476	0.2756	0.084
HUN×Female	-0.856	0.2987	0.004
ITA×Female	-0.418	0.1374	0.002
JPN×Female	-0.661	0.1293	0.000
KOR×Female	0.304	0.3260	0.351
NED×Female	-0.548	0.2645	0.038
POL×Female	0.473	0.2011	0.019
RUS×Female	-0.358	0.2195	0.102
SUI×Female	0.583	0.1439	0.000
SWE×Female	-1.207	0.3046	0.000
TPE×Female	-0.586	0.2747	0.033
USA×Female	0.028	0.1383	0.840

**Table 11:** Results from linear regression with truncated dataset time = sex×(year+year^2^)+sex×(age+age^2^))+sex×nationality and referenced to male, age 44, year 2009 and nationality Australia.

	Coefficient	Standard error	P-Value
Intercept	11.110	0.0427	0.000
Sex (female)	0.047	0.0969	0.628
Age	0.021	0.0004	0.000
Age squared	0.0011	0.0000	0.000
Year	0.070	0.0008	0.000
Year squared	0.0024	0.0000	0.000
Female×Age	0.007	0.0013	0.000
Female×Age squared	-0.0004	0.0001	0.000
Female×Year	0.015	0.0025	0.000
Female×Year squared	0.0000	0.0001	0.857
AUT	-0.319	0.0614	0.000
BEL	-0.705	0.0575	0.000
CAN	-0.094	0.0615	0.128
CHN	0.758	0.1064	0.000
CZE	-0.561	0.0690	0.000
DEN	-0.764	0.0730	0.000
ESP	-1.073	0.0559	0.000
FIN	-0.880	0.0636	0.000
FRA	0.058	0.0438	0.182
GBR	-0.740	0.0589	0.000
GER	-0.316	0.0441	0.000
HKG	0.787	0.1674	0.000
HUN	-1.241	0.0762	0.000
ITA	0.515	0.0443	0.000
JPN	0.261	0.0431	0.000
KOR	0.579	0.0595	0.000
NED	-0.553	0.0596	0.000
POL	-0.137	0.0522	0.009
RUS	-2.124	0.0616	0.000
SUI	0.242	0.0449	0.000
SWE	-0.620	0.0759	0.000
TPE	0.678	0.0545	0.000
USA	0.353	0.0484	0.000
AUT×Female	0.222	0.1713	0.195
BEL×Female	0.148	0.1704	0.386
CAN×Female	0.393	0.1282	0.002
CHN×Female	0.127	0.3268	0.698
CZE×Female	0.450	0.1899	0.018
DEN×Female	0.436	0.1792	0.015
ESP×Female	0.693	0.1996	0.001
FIN×Female	0.754	0.1505	0.000
FRA×Female	0.304	0.1015	0.003
GBR×Female	-0.434	0.1321	0.001
GER×Female	0.521	0.1015	0.000
HKG×Female	-0.137	0.4468	0.759
HUN×Female	0.068	0.1701	0.691
ITA×Female	0.134	0.1040	0.196
JPN×Female	0.164	0.0979	0.094
KOR×Female	0.022	0.2418	0.928
NED×Female	0.385	0.1582	0.015
POL×Female	0.600	0.1476	0.000
RUS×Female	0.534	0.1325	0.000
SUI×Female	0.536	0.1085	0.000
SWE×Female	0.051	0.1715	0.767
TPE×Female	-0.158	0.1617	0.327
USA×Female	0.108	0.1081	0.317

**Table 12:** Results from truncated regression with truncated dataset time = sex×(year+year^2^)+sex×(age+age^2^) + sex×nationality and referenced to male, age 44, year 2009 and nationality Australia.

	Coefficient	Standard error	P-Value
Intercept	11.490	0.067	0.000
Sex (female)	0.003	0.153	0.985
Age	0.038	0.001	0.000
Age squared	0.002	0.000	0.000
Year	0.109	0.001	0.000
Year squared	0.004	0.000	0.000
Female×Age	0.017	0.002	0.000
Female×Age squared	-0.000	0.000	0.123
Female×Year	0.035	0.004	0.000
Female×Year squared	0.001	0.000	0.010
AUT	-0.443	0.093	0.000
BEL	-0.912	0.086	0.000
CAN	-0.087	0.095	0.358
CHN	1.672	0.215	0.000
CZE	-0.717	0.102	0.000
DEN	-0.985	0.107	0.000
ESP	-1.343	0.083	0.000
FIN	-1.138	0.094	0.000
FRA	0.109	0.069	0.115
GBR	-0.935	0.088	0.000
GER	-0.405	0.069	0.000
HKG	1.454	0.330	0.000
HUN	-1.538	0.109	0.000
ITA	0.856	0.070	0.000
JPN	0.497	0.068	0.000
KOR	1.226	0.106	0.000
NED	-0.698	0.090	0.000
POL	-0.174	0.081	0.031
RUS	-2.464	0.089	0.000
SUI	0.389	0.071	0.000
SWE	-0.933	0.112	0.000
TPE	1.390	0.096	0.000
USA	0.614	0.078	0.000
AUT×Female	0.377	0.259	0.146
BEL×Female	0.676	0.255	0.008
CAN×Female	0.625	0.201	0.002
CHN×Female	0.245	0.701	0.726
CZE×Female	0.496	0.284	0.081
DEN×Female	0.672	0.268	0.012
ESP×Female	0.871	0.296	0.003
FIN×Female	0.852	0.232	0.000
FRA×Female	0.570	0.161	0.000
GBR×Female	-0.414	0.196	0.034
GER×Female	0.858	0.160	0.000
HKG×Female	-0.442	0.839	0.599
HUN×Female	0.267	0.242	0.270
ITA×Female	0.364	0.167	0.029
JPN×Female	0.368	0.156	0.018
KOR×Female	0.074	0.468	0.875
NED×Female	0.613	0.243	0.011
POL×Female	1.219	0.241	0.000
RUS×Female	0.699	0.194	0.000
SUI×Female	0.950	0.173	0.000
SWE×Female	0.311	0.258	0.228
TPE×Female	-0.190	0.294	0.517
USA×Female	0.218	0.173	0.208

**Table 13:** Interaction with race site, results from truncated regression with complete data set time = sex×(year+year^2^)+sex×(age+age^2^) + sex×nationality×site and referenced to male, age 44, year 2009 and nationality Austria.

	Coefficient	Standard error	P-Value
Intercept	14.043	0.0615	0.000
Sex (female)	1.029	0.1296	0.000
Age	0.013	0.0006	0.000
Age squared	0.0032	0.0000	0.000
Year	0.157	0.0013	0.000
Year squared	0.0062	0.0000	0.000
	0.157	0.0013	0.000
Female×Age	0.021	0.0019	0.000
Female×Age squared	-0.0007	0.0001	0.000
Female×Year	0.009	0.0039	0.015
Female×Year squared	0.0016	0.0001	0.000
AUT	-3.621	0.1796	0.000
BEL	-3.480	0.1234	0.000
CAN	-1.300	0.1076	0.000
CHN	5.201	0.0869	0.000
CZE	2.448	0.1055	0.000
DEN	-3.661	0.1475	0.000
ESP	-0.399	0.0851	0.000
FIN	-3.773	0.1179	0.000
FRA	-1.332	0.0637	0.000
GBR	-2.477	0.1188	0.000
GER	-2.968	0.0673	0.000
HKG	4.780	0.1187	0.000
HUN	-3.101	0.1529	0.000
ITA	-0.511	0.0643	0.000
JPN	-2.961	0.0626	0.000
KOR	-1.707	0.0907	0.000
NED	-3.523	0.1151	0.000
POL	0.632	0.0801	0.000
RUS	-4.060	0.1229	0.000
SUI	-0.424	0.0656	0.000
SWE	-3.181	0.1519	0.000
TPE	-2.170	0.0910	0.000
USA	-0.012	0.0693	0.867
AUT×race abroad	3.994	0.2913	0.000
BEL×race abroad	4.684	0.2605	0.000
CAN×race abroad	2.645	0.2770	0.000
CHN×race abroad	1.420	0.2545	0.000
CZE×race abroad	-2.328	0.2665	0.000
DEN×race abroad	4.545	0.3366	0.000
ESP×race abroad	2.878	0.2568	0.000
FIN×race abroad	1.972	0.3570	0.000
FRA×race abroad	4.657	0.2278	0.000
GBR×race abroad	4.787	0.2561	0.000
GER×race abroad	3.308	0.2240	0.000
HKG×race abroad	-0.484	0.4050	0.232
HUN×race abroad	1.293	0.3396	0.000
ITA×race abroad	1.596	0.2406	0.000
JPN×race abroad	5.831	0.2636	0.000
KOR×race abroad	5.142	0.4134	0.000
NED×race abroad	4.362	0.2723	0.000
POL×race abroad	-0.624	0.2490	0.012
RUS×race abroad	0.267	0.2873	0.353
SUI×race abroad	2.078	0.2632	0.000
SWE×race abroad	3.296	0.3443	0.000
TPE×race abroad	2.539	0.3055	0.000
USA×race abroad	1.300	0.2495	0.000
AUT×female	-1.319	0.5884	0.025
BEL×female	-0.744	0.4024	0.064
CAN×female	0.792	0.2117	0.000
CHN×female	-0.483	0.2139	0.024
CZE×female	0.357	0.2593	0.169
DEN×female	-0.611	0.3860	0.113
ESP×female	0.849	0.2644	0.001
FIN×female	-0.351	0.2769	0.205
FRA×female	-0.068	0.1385	0.626
GBR×female	-0.251	0.2474	0.310
GER×female	-0.913	0.1526	0.000
HKG×female	0.650	0.2918	0.026
HUN×female	-1.180	0.4018	0.003
ITA×female	-0.491	0.1413	0.001
JPN×female	-0.781	0.1331	0.000
KOR×female	-0.311	0.3419	0.363
NED×female	-0.730	0.3210	0.023
POL×female	0.025	0.2143	0.906
RUS×female	-0.436	0.2716	0.109
SUI×female	0.442	0.1477	0.003
SWE×female	-1.042	0.3398	0.002
TPE×female	-0.522	0.3117	0.094
USA×female	0.092	0.1427	0.518
AUT× race abroad×female	-1.319	0.5884	0.092
BEL× race abroad×female	-0.744	0.4024	0.043
CAN× race abroad×female	0.792	0.2117	0.053
CHN× race abroad×female	-0.483	0.2139	0.000
CZE× race abroad×female	0.357	0.2593	0.291
DEN× race abroad×female	-0.611	0.3860	0.771
ESP× race abroad×female	0.849	0.2644	0.000
FIN× race abroad×female	-0.351	0.2769	0.000
FRA× race abroad×female	-0.068	0.1385	0.556
GBR× race abroad×female	-0.251	0.2474	0.335
GER× race abroad×female	-0.913	0.1526	0.004
HKG× race abroad×female	0.650	0.2918	0.982
HUN× race abroad×female	-1.180	0.4018	0.030
ITA× race abroad×female	-0.491	0.1413	0.356
JPN× race abroad×female	-0.781	0.1331	0.004
KOR× race abroad×female	-0.311	0.3419	0.011
NED× race abroad×female	-0.730	0.3210	0.189
POL× race abroad×female	0.025	0.2143	0.000
RUS× race abroad×female	-0.436	0.2716	0.031
SUI× race abroad×female	0.442	0.1477	0.005
SWE× race abroad×female	-1.042	0.3398	0.816
TPE× race abroad×female	-0.522	0.3117	0.870
USA× race abroad×female	0.092	0.1427	0.005

**Table 14:** Interaction with race site, results from linear regression with truncated dataset time = sex×year+year^2^)+sex×(age+age^2^) + sex×nationality×site and referenced to male, age 44, year 2009, site at home and nationality Australia.

	Coefficient	Standard error	P-Value
Intercept	11.319	0.0447	0.000
Sex (female)	0.139	0.1045	0.182
Age	0.020	0.0004	0.000
Age squared	0.0011	0.0000	0.000
Year	0.068	0.0008	0.000
Year squared	0.0024	0.0000	0.000
Female×Age	0.005	0.0013	0.000
Female×Age squared	-0.0004	0.0001	0.000
Female×Year	0.006	0.0025	0.013
Female×Year squared	-0.0002	0.0001	0.152
AUT	-1.092	0.0953	0.000
BEL	-1.228	0.0701	0.000
CAN	-0.128	0.0668	0.056
CHN	0.601	0.1196	0.000
CZE	-0.314	0.0984	0.001
DEN	-0.831	0.0792	0.000
ESP	-1.188	0.0601	0.000
FIN	-0.994	0.0667	0.000
FRA	-0.136	0.0458	0.003
GBR	-1.322	0.0737	0.000
GER	-1.040	0.0472	0.000
HKG	0.841	0.2060	0.000
HUN	-0.986	0.0867	0.000
ITA	0.336	0.0463	0.000
JPN	0.074	0.0451	0.102
KOR	0.432	0.0609	0.000
NED	-0.856	0.0658	0.000
POL	-0.105	0.0567	0.063
RUS	-1.672	0.0706	0.000
SUI	0.001	0.0469	0.986
SWE	-0.661	0.0835	0.000
TPE	0.510	0.0568	0.000
USA	0.273	0.0507	0.000
AUT×race abroad	2.484	0.1627	0.000
BEL×race abroad	2.343	0.1501	0.000
CAN×race abroad	0.957	0.1663	0.000
CHN×race abroad	1.571	0.2566	0.000
CZE×race abroad	1.002	0.1705	0.000
DEN×race abroad	1.025	0.1950	0.000
ESP×race abroad	1.297	0.1558	0.000
FIN×race abroad	0.860	0.1944	0.000
FRA×race abroad	1.484	0.1350	0.000
GBR×race abroad	2.418	0.1524	0.000
GER×race abroad	2.584	0.1308	0.000
HKG×race abroad	1.065	0.3543	0.003
HUN×race abroad	0.197	0.1876	0.295
ITA×race abroad	0.735	0.1427	0.000
JPN×race abroad	-0.399	0.1659	0.016
KOR×race abroad	0.012	0.2824	0.965
NED×race abroad	2.018	0.1583	0.000
POL×race abroad	0.864	0.1450	0.000
RUS×race abroad	-0.102	0.1583	0.520
SUI×race abroad	0.884	0.1622	0.000
SWE×race abroad	1.021	0.1956	0.000
TPE×race abroad	1.460	0.1714	0.000
USA×race abroad	0.567	0.1488	0.000
AUT×female	-0.564	0.3079	0.067
BEL×female	0.207	0.2213	0.349
CAN×female	0.324	0.1445	0.025
CHN×female	0.293	0.3774	0.437
CZE×female	-0.191	0.2767	0.489
DEN×female	0.408	0.2037	0.045
ESP×female	0.377	0.2155	0.081
FIN×female	0.567	0.1591	0.000
FRA×female	0.296	0.1089	0.007
GBR×female	-0.032	0.1626	0.845
GER×female	0.371	0.1127	0.001
HKG×female	0.798	0.8050	0.321
HUN×female	-0.139	0.2253	0.536
ITA×female	0.161	0.1113	0.149
JPN×female	0.144	0.1053	0.171
KOR×female	0.088	0.2539	0.729
NED×female	0.278	0.1813	0.125
POL×female	0.191	0.1639	0.244
RUS×female	0.284	0.1617	0.079
SUI×female	0.454	0.1155	0.000
SWE×female	0.024	0.1890	0.897
TPE×female	-0.121	0.1845	0.512
USA×female	0.234	0.1167	0.045
AUT× race abroad×female	0.658	0.4180	0.115
BEL× race abroad×female	-0.506	0.3768	0.179
CAN× race abroad×female	-0.052	0.3147	0.868
CHN× race abroad×female	-0.906	0.7296	0.214
CZE× race abroad×female	0.692	0.4175	0.098
DEN× race abroad×female	-0.199	0.4214	0.637
ESP× race abroad×female	1.056	0.5267	0.045
FIN× race abroad×female	0.958	0.4499	0.033
FRA× race abroad×female	-1.505	0.2853	0.000
GBR× race abroad×female	-1.157	0.3115	0.000
GER× race abroad×female	-0.119	0.2609	0.648
HKG× race abroad×female	-1.186	0.9940	0.233
HUN× race abroad×female	0.672	0.3816	0.078
ITA× race abroad×female	-1.355	0.3009	0.000
JPN× race abroad×female	-0.214	0.3048	0.482
KOR× race abroad×female	0.116	0.7603	0.878
NED× race abroad×female	-0.144	0.3698	0.697
POL× race abroad×female	0.900	0.3611	0.013
RUS× race abroad×female	0.585	0.3127	0.061
SUI× race abroad×female	-0.592	0.3508	0.091
SWE× race abroad×female	-0.452	0.4260	0.289
TPE× race abroad×female	-0.285	0.3870	0.461
USA× race abroad×female	-0.878	0.2887	0.002

**Table 15:** Comparing times in hours with finishes performed. Times were computed based on the model time = sex×(year+year^2^)+sex×(age+age^2^) + sex×nationality and referenced to male, age 44, year 2009 and nationality Australia. In parentheses: 95%-CI.

	A		B		% Difference	C	
	Data: complete Linear regression with fixed effects		Data: truncated at 15 hours Linear regression with fixed effects		Between A and B (B-A)/A	Data: truncated at 15 hours Truncated linear regression with fixed effects	
AUS	13.9	(13.8–14.0)	11.1	(11.0–11.2)	-20.0%	11.5	(11.4–11.6)
AUT	12.0	(11.7–12.2)	10.8	(10.6–10.9)	-9.8%	11.0	(10.8–11.3)
BEL	12.2	(12.0–12.4)	10.4	(10.3–10.5)	-14.5%	10.6	(10.4–10.8)
CAN	12.9	(12.7–13.1)	11.0	(10.9–11.2)	-14.6%	11.4	(11.2–11.6)
CHN	19.1	(18.9–19.3)	11.9	(11.6–12.1)	-37.8%	13.2	(12.7–13.6)
CZE	15.0	(14.8–15.2)	10.5	(10.4–10.7)	-29.6%	10.8	(10.5–11.0)
DEN	11.1	(10.8–11.4)	10.3	(10.2–10.5)	-6.6%	10.5	(10.3–10.8)
ESP	13.8	(13.6–14.0)	10.0	(9.9–10.2)	-27.3%	10.1	(9.9–10.4)
FIN	10.2	(10.0–10.5)	10.2	(10.1–10.4)	-0.1%	10.4	(10.1–10.6)
FRA	13.0	(12.8–13.2)	11.2	(11.0–11.3)	-14.0%	11.6	(11.4–11.8)
GBR	13.3	(13.1–13.5)	10.4	(10.2–10.5)	-21.9%	10.6	(10.3–10.8)
GER	11.8	(11.6–12.0)	10.8	(10.7–10.9)	-8.6%	11.1	(10.9–11.3)
HKG	18.6	(18.3–18.8)	11.9	(11.6–12.2)	-36.0%	12.9	(12.3–13.6)
HUN	10.7	(10.4–11.0)	9.9	(9.7–10.0)	-7.8%	10.0	(9.7–10.2)
ITA	13.5	(13.3–13.7)	11.6	(11.5–11.7)	-13.9%	12.3	(12.2–12.5)
JPN	11.1	(10.9–11.3)	11.4	(11.3–11.5)	2.3%	12.0	(11.8–12.2)
KOR	12.5	(12.2–12.7)	11.7	(11.5–11.8)	-6.1%	12.7	(12.5–13.0)
NED	11.4	(11.2–11.6)	10.6	(10.4–10.7)	-7.2%	10.8	(10.6–11.0)
POL	14.1	(13.9–14.3)	11.0	(10.8–11.1)	-22.2%	11.3	(11.1–11.5)
RUS	9.4	(9.1–9.6)	9.0	(8.8–9.1)	-3.9%	9.0	(8.8–9.2)
SUI	13.6	(13.4–13.7)	11.4	(11.2–11.5)	-16.3%	11.9	(11.7–12.1)
SWE	11.2	(10.9–11.5)	10.5	(10.3–10.7)	-6.3%	10.6	(10.3–10.8)
TPE	11.9	(11.7–12.1)	11.8	(11.7–11.9)	-1.1%	12.9	(12.7–13.1)
USA	14.0	(13.8–14.1)	11.5	(11.3–11.6)	-17.9%	12.1	(11.9–12.3)

**Table 16:** Comparing times in hours with finishes performed at home. Times were computed based on the model time = ex×(year+year^2^)+sex×(age+age^2^) + sex×nationality×site and referenced to male, age 44, year 2009 and nationality Australia.

	A		% Difference between races abroad/at home	B		% Difference between races at home/on abroad	C		% Difference between races abroad/at home
	Data: complete Linear regression with fixed effects			Data: truncated at 14 hours Linear regression with fixed effects			Data: truncated at 14 hours Truncated linear regression with fixed effects		
	Races at home	Races abroad		Races at home	Races abroad		Races at home	Races abroad	
AUS	14.0	12.0	11.3	11.3	9.6	-15.4%	11.7	9.6	-17.8%
AUT	10.4	12.4	10.2	10.2	11.0	7.2%	10.5	11.4	9.3%
BEL	10.6	13.2	10.1	10.1	10.7	5.9%	10.2	10.9	6.5%
CAN	12.7	13.3	11.2	11.2	10.4	-7.1%	11.7	10.6	-9.4%
CHN	19.2	18.6	11.9	11.9	11.7	-1.5%	13.1	13.2	0.7%
CZE	16.5	12.1	11.0	11.0	10.3	-6.8%	11.3	10.3	-8.8%
DEN	10.4	12.9	10.5	10.5	9.8	-6.9%	10.7	9.9	-7.2%
ESP	13.6	14.5	10.1	10.1	9.7	-4.4%	10.3	10.0	-3.2%
FIN	10.3	10.2	10.3	10.3	9.4	-8.6%	10.4	9.3	-10.6%
FRA	12.7	15.3	11.2	11.2	10.9	-2.4%	11.6	11.2	-3.4%
GBR	11.6	14.3	10.0	10.0	10.7	6.7%	10.1	10.9	8.1%
GER	11.1	12.3	10.3	10.3	11.1	8.1%	10.4	11.5	10.6%
HKG	18.8	16.3	12.2	12.2	11.5	-5.6%	13.1	11.2	-14.5%
HUN	10.9	10.2	10.3	10.3	8.8	-15.0%	10.5	8.9	-15.3%
ITA	13.5	13.1	11.7	11.7	10.6	-8.7%	12.4	10.8	-12.9%
JPN	11.1	14.9	11.4	11.4	9.2	-18.8%	12.0	9.4	-21.4%
KOR	12.3	15.4	11.8	11.8	10.0	-14.8%	12.8	10.3	-19.4%
NED	10.5	12.8	10.5	10.5	10.7	2.6%	10.6	11.0	4.0%
POL	14.7	12.0	11.2	11.2	10.3	-7.9%	11.6	10.5	-9.2%
RUS	10.0	8.2	9.6	9.6	7.8	-19.2%	9.5	7.9	-17.2%
SUI	13.6	13.7	11.3	11.3	10.5	-7.6%	11.8	10.4	-12.3%
SWE	10.9	12.1	10.7	10.7	9.9	-6.8%	10.7	9.8	-8.5%
TPE	11.9	12.4	11.8	11.8	11.5	-2.4%	12.9	12.2	-5.8%
USA	14.0	13.3	11.6	11.6	10.4	-10.2%	12.4	10.6	-14.1%

The upper panel of Fig 9(A)–9(C) display the time for each nationality and confidence intervals from the multivariable model (1) at reference sex = male, age = 44 and year = 2009. (A) was computed with the complete data set, (B) with the truncated data set and (C) was performed by truncated regression and truncated data set. The ranks from the results with complete data set show that runners from Russia, Finland, and Hungary were the fastest and runners from Hong Kong and China were the slowest finishers. The ranks from the results from the truncated dataset changed to Russia, Hungary, Spain and Great Britain.

To visualize changes in ranks between these three methods the estimates were ordered by descending time estimates and the nationality of the estimates were connected with lines. Fig 9(D) shows the changes between results from linear regression with complete data set and truncated data set and Fig 9(E) shows the results from linear regression with the truncated data set and truncated regression. Comparing the regression of complete with truncated data set shows that Russia, Canada, Hong Kong and China hold their position where all other nationalities change their rank. Japan changed from rank 5 to rank 18. Hungary changed from third to second and Finland from second to fourth. The time for Russia changed from 9.4 h (95%-CI: 9.1–9.6) to 9.0 h (95%-CI: 8.9–9.1), Hungary from 10.7 h (95%-CI 10.4–11.0) to 9.9 h (95%-CI: 9.7–10.0), Japan from 11.1 h (95%-CI: 10.9–11.3) to 11.4 h (95%-CI: 11.3–11.5) and China from 19.1 h (95%-CI: 18.9–19.3) to 11.9 h (95%-CI: 11.6–12.1).

There are only four nationalities which change ranks when ranks from linear regression are compared with ranks from truncated regression both based on truncated dataset (Fig 9E). Fig 10(A) and 10(B) shows changes of ranks between running at home and abroad computed with complete data set, respectively, truncated data set based on model (2). Both show many changes in rank position. Again Russia remains at place 1 at home and abroad with 10.0 h and 8.2 h, respectively, with complete data set and 9.7 h and 7.8 with truncated data set. Japan changed from rank 10 (11.1 h, at home) to rank 20 (14.9 h, abroad) when complete dataset was used and from rank 18 (11.4 h) to 3 (9.2 h) when truncated dataset was used (Table 16).

Table 17 shows the mean time of the top 10, 100 and 1000 finishers. Only the fastest finishes of each finisher was considered to define the top. Japan is first at top 10 and top 100 and second at top 1000 whereas Russia is second at top 10 and top 100 and ninth at top 1000. Table 18 shows the mean time of the top fastest finishes. Russia is first at top 10 and 100 with 5 finisher and 10 finishes and 37 finishers with 100 finishes. Japan is second at top 10 and 100 with 7 finishers and 10 finishes and 50 finishes with 100 finishes.

**Table 17:** Mean time of the top 10, 100 and 1000 of the fastest finishers for each nationality. Only the lowest time of a finishes was considered if one finisher had several finishes.

	Nationality	N of finishers	Mean time of top 10	Nationality	N of finishers	Mean time of top 100	Nationality	N of finishers	Mean time of top 1000
1	JPN	10	6.41	JPN	100	6.83	GER	1000	7.83
2	RUS	10	6.43	RUS	100	6.86	JPN	1000	7.97
3	FRA	10	6.52	GER	100	7.00	FRA	1000	8.19
4	GBR	10	6.55	FRA	100	7.01	SUI	1000	8.31
5	ESP	10	6.61	SUI	100	7.18	ITA	1000	8.81
6	BEL	10	6.62	GBR	100	7.29	USA	1000	9.46
7	USA	10	6.62	USA	100	7.31	POL	1000	10.24
8	GER	10	6.63	ITA	100	7.38	GBR	1000	10.80
9	POL	10	6.65	ESP	100	7.57	RUS	920	10.82
10	ITA	10	6.67	BEL	100	7.74	FIN	479	10.96
11	SUI	10	6.72	POL	100	7.96	ESP	1000	11.05
12	FIN	10	6.79	NED	100	8.06	TPE	1000	11.42
13	CZE	10	6.81	AUS	100	8.08	AUS	1000	11.60
14	HUN	10	6.81	HUN	100	8.18	DEN	544	11.62
15	NED	10	6.87	AUT	100	8.21	HUN	419	11.62
16	SWE	10	6.89	FIN	100	8.28	CAN	1000	11.86
17	AUS	10	6.95	CZE	100	8.34	NED	805	12.10
18	DEN	10	7.33	CAN	100	8.38	BEL	1000	12.14
19	AUT	10	7.34	SWE	100	8.40	SWE	570	12.18
20	CAN	10	7.36	DEN	100	8.61	KOR	1000	12.37
21	KOR	10	7.68	KOR	100	9.05	AUT	969	12.66
22	TPE	10	7.99	TPE	100	9.26	CZE	1000	14.81
23	CHN	10	8.85	CHN	100	11.14	CHN	1000	15.31
24	HKG	10	9.47	HKG	100	12.41	HKG	1000	19.22

**Table 18:** Mean time of the top 10, 100 and 1000 of the fastest finishes for each nationality.

	Nationality	N of finishers	Mean time of top 10	Nationality	N of finishers	Mean time of top 100	Nationality	N of finishers	Mean time of top 1000
1	RUS	5	6.37	RUS	37	6.57	GER	332	7.35
2	JPN	7	6.37	JPN	50	6.68	FRA	358	7.52
3	BEL	3	6.44	FRA	35	6.74	JPN	557	7.60
4	POL	3	6.47	GER	34	6.80	RUS	351	7.70
5	GBR	7	6.47	GBR	30	6.86	SUI	393	7.74
6	ITA	4	6.49	BEL	23	6.88	ITA	362	8.02
7	FRA	7	6.50	ITA	30	6.94	GBR	402	8.54
8	ESP	4	6.50	ESP	28	6.96	USA	543	8.58
9	GER	5	6.54	SUI	47	6.96	ESP	365	8.60
10	CZE	3	6.55	USA	42	6.99	BEL	359	8.87
11	USA	7	6.60	POL	25	7.00	POL	503	9.25
12	SUI	4	6.68	HUN	29	7.25	NED	415	9.49
13	SWE	5	6.68	NED	29	7.32	FIN	358	9.83
14	HUN	5	6.70	CZE	24	7.33	CAN	463	9.94
15	FIN	7	6.72	AUS	42	7.55	AUT	532	10.04
16	NED	4	6.77	FIN	40	7.64	AUS	536	10.14
17	AUS	4	6.81	SWE	47	7.69	CZE	451	10.82
18	CAN	3	6.89	CAN	41	7.80	TPE	753	11.05
19	DEN	7	7.26	AUT	47	7.86	HUN	419	11.18
20	AUT	6	7.28	DEN	47	8.11	DEN	544	11.59
21	KOR	5	7.42	KOR	72	8.76	KOR	625	11.62
22	TPE	9	7.98	TPE	79	9.15	SWE	570	11.96
23	CHN	9	8.85	CHN	84	10.95	CHN	808	14.82
24	HKG	10	9.47	HKG	90	12.21	HKG	846	18.46

## Discussion

The aim of this study was to investigate the aspect of nationality of the fastest 100-km ultra-marathons competing between 1959 and 2016 with the hypothesis that the fastest runners would originate from Japan as it has been found for the top 10 runners worldwide competing between 1998 and 2011. However, in contrast to previous findings, athletes from Russia achieved the fastest race times, not athletes from Japan, when all athletes were considered by nationality.

A first potential explanation could be the quote of finishes at the origin country. For example, Russians have ~37% of the finishes outside the origin but Japanese less than 2%. Most probably only the fastest Russian runners compete outside Russia on the fastest races (*e*.*g*. World Championships) or the fastest courses (*e*.*g*. completely flat course, track races) worldwide. In contrast, Japanese runners competed preferably in races held in Japan where the courses are most probably not fast (*i*.*e*. rather hilly courses than flat courses). The present study is, however, not the first to show that Russian athletes are the fastest in an ultra-endurance sport. Recently, an analysis of the ‘Engadin Ski Marathon’ showed that Russians were the fastest cross-country skiers [11], which, combined with the findings of the present study, indicated a general trend of excellence of Russians in ultra-endurance sports.

The two strongest factors which seems influence the population speed are the attitude to participate and rules concerning time limits. Firstly, as the density plots and histograms show it seems that there are countries where also very slow participants were allowed to compete in races and who has been also considered in the ranking. Extreme examples of this kind of competitions are athletes from the nationalities China, and Hong Kong, but also Czech Republic, Great Britain, Spain, and Australia. Athletes from other countries like Denmark, Finland, Sweden, and Russia have a high skewness and excess which means that the bunch is concentrated over a narrow limit. This may due to attitudes within society (*e*.*g*. popularity of sports, policy of furtherance) or socio-economic backgrounds of the individuals that only fast competitors participates. It has been suggested also that a successful finish in this sport depends on the motivation to train intensively [7].

Secondly, the density plots of athletes from Japan, Taiwan and Korea show a very steep slope on the right side of the curve. This may due to time limits given by the organizer. These time limits may not only performed in Japan or Taiwan but less frequently also in other countries. This could be the main source of bias which would explain why Japanese were the fastest [1]. To counteract this bias we truncated the dataset and considered only finishes with lower or equal 14 hours. This can cause bias like using top 10 finishers if we would conclude to the whole population. So, we have to consider that using the complete dataset would give bias due to policy rules and if we use the truncated dataset we have selection bias. We used also truncated regression to allow conclusion to the whole running population but it seems that too many observations have been truncated which changed the dataset in that way that it changed completely the shape of the original distribution which does not anymore allow conclusion on the complete data set but only on the truncated data set. That’s why the linear regression and truncated regression of the truncated data set gives similar results. Nonetheless, the assumption that in Japan is a time limit may be supported by the fact that the race time at home is 11.1 h and on abroad is 14.9 h using model (2) and complete data set. This is an increase of 3.1 hours. For athletes from Russia, the times are 10.0 h and 8.2 h, respectively, a decrease of 1.7 hours. Assuming that only good and the best ultra-marathoners take the effort to go abroad the mean time should decrease which is not the case for Japanese runners. Nevertheless, both analyses with the complete and the truncated dataset show that Russian runners were the fastest and athletes from China and Hong Kong were the slowest. All other nationalities change their rankings reflecting the distribution of the running time.

If we look at the top 10, 100 and 1000 of the fastest finishers, Japanese ultra-marathoners take the first place and the second place, respectively. The rank shifts to the rear the more participants are included in the data set. It seems that there some very fast Japanese but as the number of participants growths the mean time increase more than in other nationalities. A look at the top 10, 100 of the finishes shows that Russian ultra-marathoners take the first places. This is due to five runners with 10 finishes and 37 runners with 100 finishes. In this case it seems that Russian ultra-marathoners take high ranks since some few runners achieved some very fast finishes. This could be a limit of the linear regression if finishers are not considered in a multilevel regression as random variable. We also performed a linear regression with finisher as random variable and we got similar results as in the linear regression with complete and truncated data set (data not shown).

The role of nationality on 100-km ultra-marathon race times highlighted in the present study was in agreement with previous research that identified sports as the most powerful form of national performance [12]. An attempt to use sport to build a sense of national identity has been reported [13]. Either biological or cultural heredity has been identified as a factor associated with the dominance of a nationality in a sport [14]. For instance, certain genes have been identified to relate to endurance performance [15]. In addition, an explanation of the differences in participation among nationalities might be the differences in their attitudes towards physical activity [16]. Participation in running might be influenced by economic and cultural factors, *e*.*g*. those without a migration background are more likely to participate in running than those with a migration background [17]. Another aspect affecting sport performance is doping, for which no accurate rates exist due to its undisclosed practice; however, its prevalence has been estimated as 14–39% in adult elite athletes and has been shown to vary by performance level and nationality [18].

Since it has been shown that the role of nationality might vary by distance in endurance and ultra-endurance running [9], the findings of the present study should not be generalized to other distances. On the other hand, strength of the study was its methodological approach to the research question: first, it used one of the larger samples of 100-km ultra-marathoners ever studied, second, it considered a probably applied time limit barrier by using a truncated data set, third, we compared adjusted times of a reference finisher to compare ranks, and forth, we provide a time distribution classification which helps to interpret the results.

We found that ultra-marathoners from Russia were the fastest in this specific ultra-marathon distance. Unfortunately, this kind of analysis is not able to explain the reason for this dominance. The role of ethnicity in running is, however, well-known for other running distances. Running best performances are dominated by a few groups of athletes including runners with West African ancestry for the sprint distances and East African runners for the long distances [9, 19]. For marathoners, East-African runners from Kenya and Ethiopia dominate since decades running distances up to the marathon [9, 20–22] while for other running distances such as the sprint distances, runners from Jamaica are dominating [23].

For elite Kenyan runners, it is well-known that they are from a distinctive environmental background in terms of geographical distribution, ethnicity and run to school [20]. Interestingly, the same findings were reported for elite Ethiopian runners, who also have a distinct environmental background in terms of geographical distribution, ethnicity, and also often run to school [21]. So for both Kenyan and Ethiopian runners both environmental and social factors are important for their athletic success. These aspects are, however, not only for East African distance runners, but also for sprinters from Jamaica of importance. It has been shown that a higher proportion of middle distance and both jump and throw athletes walked to school and travelled greater distances to school [23].

Although different anthropometric, physiological, biomechanical and training characteristics are of importance for the East African running dominance [22, 24, 25], a strong psychological motivation to succeed athletically for the purpose of economic and social advancement is known [26]. Elite Kenyan runners become a competitive athlete due to economic reasons. Typically, Kenyan athletes see athletics as a means of making money to help their families, parents and siblings [20, 27].

### Practical applications for athletes and coaches

The present study advances existing theoretical knowledge as scientists will improve their understanding of participation and performance trends by nationality in 100-km ultra-marathon running which is relatively less studied compared to shorter distances such as sprint and marathon distances. Moreover, coaches and runners can use the findings to optimize their preparation and participation in a 100-km ultra-marathon. Athletes from other countries than the dominating nationalities (*i*.*e*. Russia, Hungary) must be aware that they will most probably not be able to reach a world-class level in 100-km ultra-marathon running.

## Conclusions

In summary, in contrast to existing findings investigating the top 10 by nationality, this analysis of all runners showed that ultra-marathoners from Russia, not Japan, were the fastest 100-km ultra-marathoners worldwide when considering all races held since 1959. Since we know for the best sprinters and marathoners in the world that specific anthropometric, training and environmental characteristics are prevalent, future studies need to investigate why Russian ultra-marathoners dominate the 100-km ultra-marathon distance.
