# Correction: Russians are the fastest 100-km ultra-marathoners in the world

**DOI:** 10.1371/journal.pone.0272170

**Published:** 2022-07-21

**Authors:** Beat Knechtle, Pantelis Theodoros Nikolaidis, Fabio Valeri

There is an error in the legend for Fig 1. Please see the correct legend for Fig 1 here.

**Fig 1:**
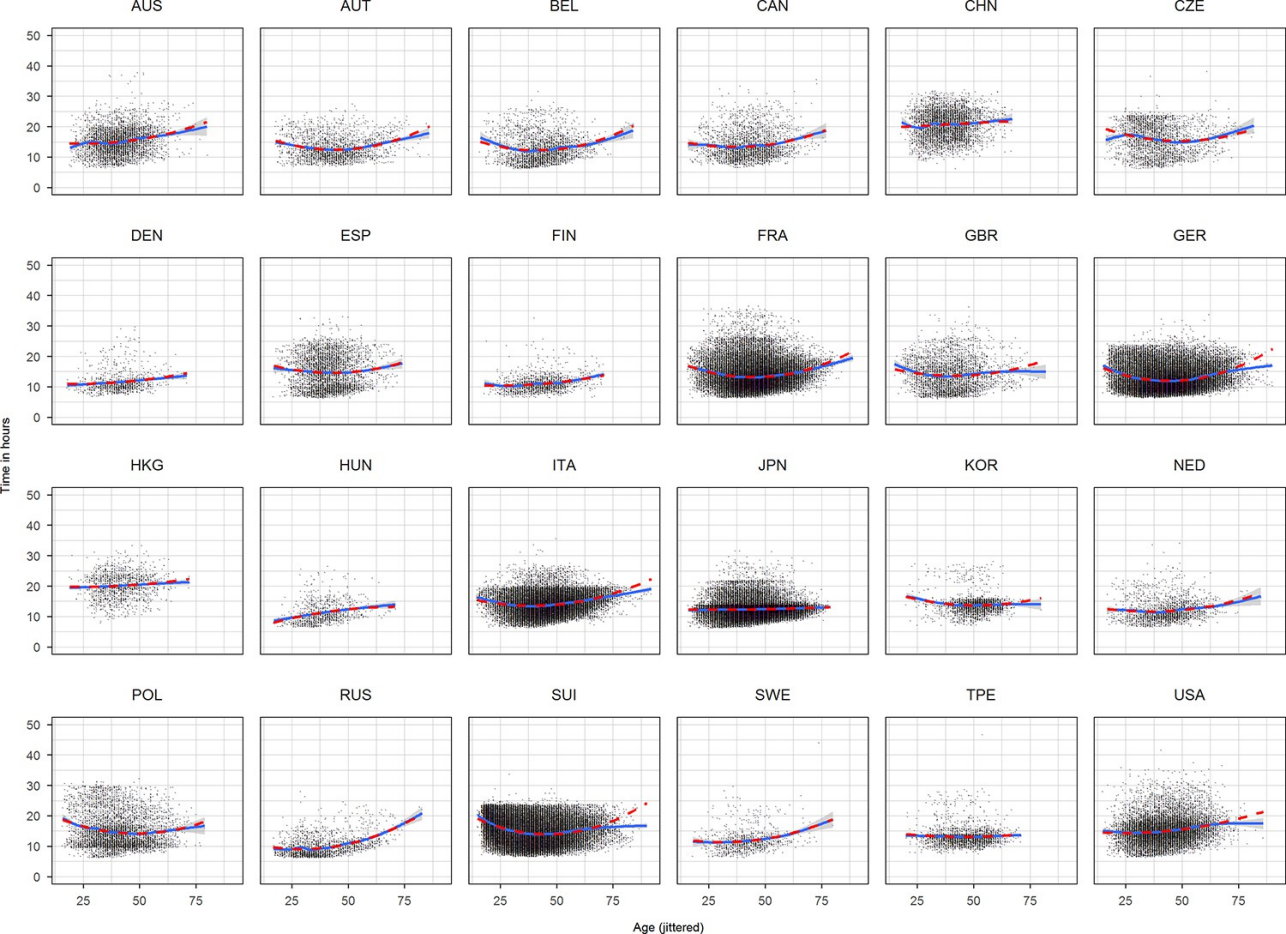
Scatterplots time against age for each nationality based on the complete dataset. Age has been jittered.

There is an error in the legend of Fig 2. Please find the correct legend for Fig 2 here.

**Fig 2:**
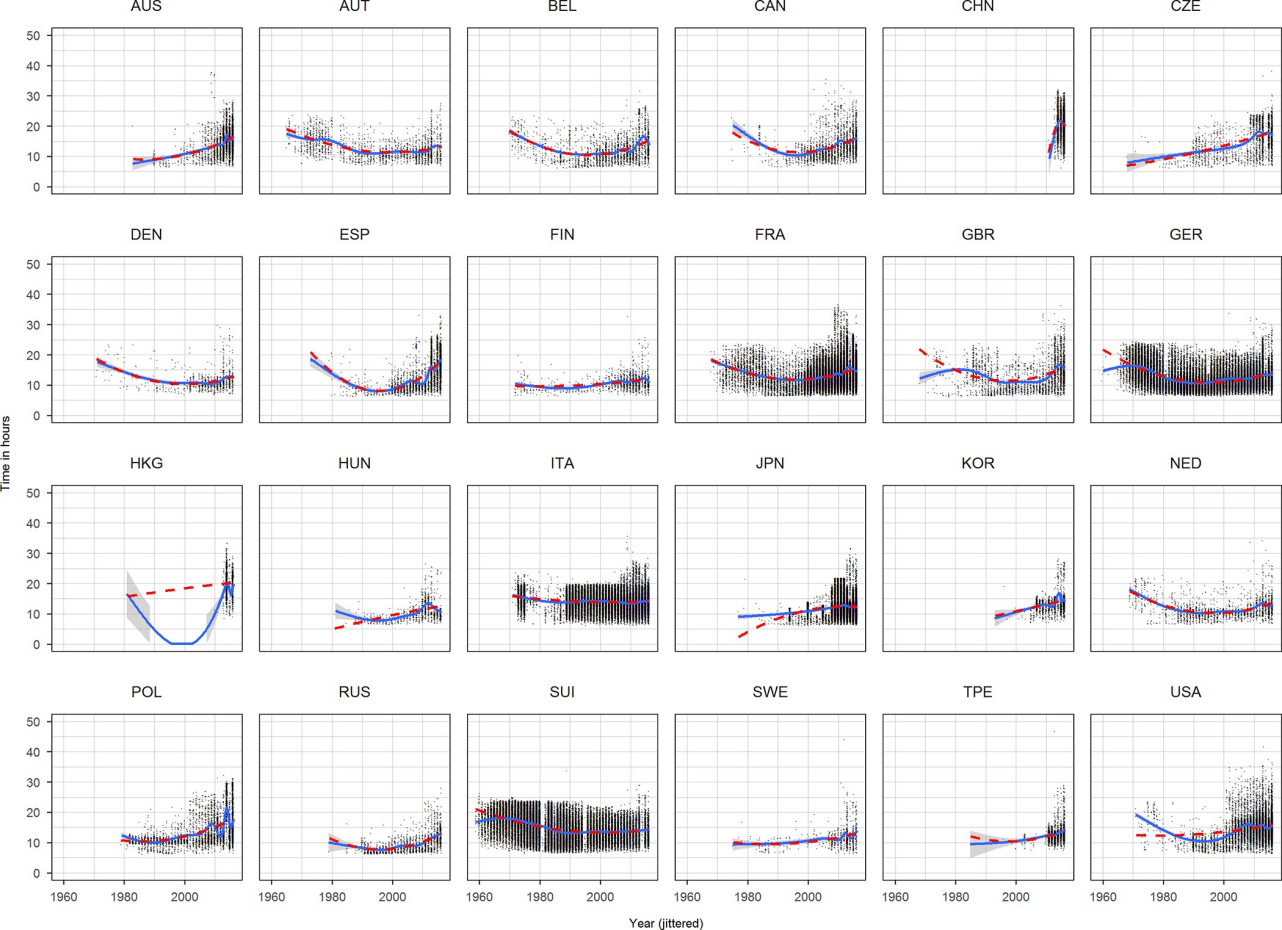
Scatterplots time against race year for each nationality based on the complete dataset. Race year has been jittered.

There is an omission of the R^2^ value in the legend of Table 10. Please see the correct legend for Table 10 here.

**Table 10:** Results from linear regression with complete dataset time = sex×(year+year^2^)+sex×(age+age^2^)+sex×nationality and referenced to male, age 44, year 2009 and nationality Australia. R^2^ = 0.26.

	Coefficent	Standard error	P-Value
Intercept	13.879	0.0600	0.000
Sex (female)	0.892	0.1254	0.000
Age	0.012	0.0006	0.000
Age squared	0.0033	0.0000	0.000
Year	0.156	0.0013	0.000
Year squared	0.0062	0.0000	0.000
FemalexAge	0.022	0.0019	0.000
FemalexAge squared	-0.0066	0.0001	0.000
FemalexYear	0.014	0.0038	0.000
FemalexYear squared	0.0016	0.0001	0.000
AUT	-1.922	0.0976	0.000
BEL	-1.713	0.0905	0.000
CAN	-0.976	0.0967	0.000
CHN	5.199	0.0808	0.000
CZE	1.104	0.0927	0.000
DEN	-2.806	0.1301	0.000
ESP	-0.069	0.0801	0.389
FIN	-3.634	0.1126	0.000
FRA	-0.898	0.0621	0.000
GBR	-0.597	0.0867	0.000
GER	-2.075	0.0634	0.000
HKG	4.708	0.1152	0.000
HUN	-3.176	0.1334	0.000
ITA	-0.378	0.0629	0.000
JPN	-2.764	0.0613	0.000
KOR	-1.425	0.0895	0.000
NED	-2.503	0.0985	0.000
POL	0.220	0.0758	0.004
RUS	-4.524	0.1064	0.000
SUI	-0.320	0.0642	0.000
SWE	-2.678	0.1339	0.000
TPE	-1.956	0.0881	0.000
USA	0.079	0.0676	0.244
AUTxFemale	-0.705	0.2708	0.009
BELxFemale	-0.245	0.2660	0.357
CANxFemale	0.330	0.1868	0.077
CHNxFemale	-0.070	0.1939	0.718
CZExFemale	0.629	0.2280	0.006
DENxFemale	-0.638	0.3222	0.048
ESPxFemale	1.444	0.2405	0.000
FINxFemale	0.149	0.2622	0.569
FRAxFemale	-0.038	0.1341	0.778
GBRxFemale	-1.098	0.1905	0.000
GERxFemale	-0.441	0.1372	0.001
HKGxFemale	0.476	0.2756	0.084
HUNxFemale	-0.856	0.2987	0.004
ITAxFemale	-0.418	0.1374	0.002
JPNxFemale	-0.661	0.1293	0.000
KORxFemale	0.304	0.3260	0.351
NEDxFemale	-0.548	0.2645	0.038
POLxFemale	0.473	0.2011	0.019
RUSxFemale	-0.358	0.2195	0.102
SUIxFemale	0.583	0.1439	0.000
SWExFemale	-1.207	0.3046	0.000
TPExFemale	-0.586	0.2747	0.033
USAxFemale	0.028	0.1383	0.840

There is an omission of the R^2^ value in the legend of Table 11. Please see the correct legend for Table 11 here.

**Table 11:** Results from linear regression with truncated dataset time = sex×(year+year^2^)+sex×(age+age^2^)+sex×nationality and referenced to male, age 44, year 2009 and nationality Australia. R^2^ = 0.17.

	Coefficient	Standard error	P-Value
Intercept	11.110	0.0427	0.000
Sex (female)	0.047	0.0969	0.628
Age	0.021	0.0004	0.000
Age squared	0.0011	0.0000	0.000
Year	0.070	0.0008	0.000
Year squared	0.0024	0.0000	0.000
Female×Age	0.007	0.0013	0.000
Female×Age squared	-0.0004	0.0001	0.000
Female×Year	0.015	0.0025	0.000
Female×Year squared	0.0000	0.0001	0.857
AUT	-0.319	0.0614	0.000
BEL	-0.705	0.0575	0.000
CAN	-0.094	0.0615	0.128
CHN	0.758	0.1064	0.000
CZE	-0.561	0.0690	0.000
DEN	-0.764	0.0730	0.000
ESP	-1.073	0.0559	0.000
FIN	-0.880	0.0636	0.000
FRA	0.058	0.0438	0.182
GBR	-0.740	0.0589	0.000
GER	-0.316	0.0441	0.000
HKG	0.787	0.1674	0.000
HUN	-1.241	0.0762	0.000
ITA	0.515	0.0443	0.000
JPN	0.261	0.0431	0.000
KOR	0.579	0.0595	0.000
NED	-0.553	0.0596	0.000
POL	-0.137	0.0522	0.009
RUS	-2.124	0.0616	0.000
SUI	0.242	0.0449	0.000
SWE	-0.620	0.0759	0.000
TPE	0.678	0.0545	0.000
USA	0.353	0.0484	0.000
AUT×Female	0.222	0.1713	0.195
BEL×Female	0.148	0.1704	0.386
CAN×Female	0.393	0.1282	0.002
CHN×Female	0.127	0.3268	0.698
CZE×Female	0.450	0.1899	0.018
DEN×Female	0.436	0.1792	0.015
ESP×Female	0.693	0.1996	0.001
FIN×Female	0.754	0.1505	0.000
FRA×Female	0.304	0.1015	0.003
GBR×Female	-0.434	0.1321	0.001
GER×Female	0.521	0.1015	0.003
HKG×Female	-0.137	0.4468	0.759
HUN×Female	0.068	0.1701	0.691
ITA×Female	0.134	0.1040	0.196
JPN×Female	0.164	0.0979	0.094
KOR×Female	0.022	0.2418	0.928
NED×Female	0.385	0.1582	0.015
POL×Female	0.600	0.1476	0.000
RUS×Female	0.534	0.1325	0.000
SUI×Female	0.536	0.1085	0.000
SWE×Female	0.051	0.1715	0.767
TPE×Female	-0.158	0.1617	0.327
USA×Female	0.108	0.1081	0.317

There is an omission of the R^2^ value in the legend of Table 12. Please see the correct legend for Table 12 here.

**Table 12:** Results from truncated regression with truncated dataset time = sex×(year+year^2^)+sex×(age+age^2^) + sex×nationality and referenced to male, age 44, year 2009 and nationality Australia. R^2^ = 0.17.

	Coefficient	Standard error	P-Value
Intercept	11.490	0.067	0.000
Sex (female)	0.003	0.153	0.985
Age	0.038	0.001	0.000
Age squared	0.002	0.000	0.000
Year	0.109	0.001	0.000
Year squared	0.004	0.000	0.000
Female×Age	0.017	0.002	0.000
Female×Age squared	-0.000	0.000	0.123
Female×Year	0.035	0.004	0.000
Female×Year squared	0.001	0.000	0.010
AUT	-0.443	0.093	0.000
BEL	-0.912	0.086	0.000
CAN	-0.087	0.095	0.358
CHN	1.672	0.215	0.000
CZE	-0.717	0.102	0.000
DEN	-0.985	0.107	0.000
ESP	-1.343	0.083	0.000
FIN	-1.138	0.094	0.000
FRA	0.109	0.069	0.115
GBR	-0.935	0.088	0.000
GER	-0.405	0.069	0.000
HKG	1.454	0.330	0.000
HUN	-1.538	0.109	0.000
ITA	0.856	0.070	0.000
JPN	0.497	0.068	0.000
KOR	1.226	0.106	0.000
NED	-0.698	0.090	0.000
POL	-0.174	0.081	0.031
RUS	-2.464	0.089	0.000
SUI	0.389	0.071	0.000
SWE	-0.933	0.112	0.000
TPE	1.390	0.096	0.000
USA	0.614	0.078	0.000
AUT×Female	0.377	0.259	0.146
BEL×Female	0.676	0.255	0.008
CAN×Female	0.625	0.201	0.002
CHN×Female	0.245	0.701	0.726
CZE×Female	0.496	0.284	0.081
DEN×Female	0.672	0.268	0.012
ESP×Female	0.871	0.296	0.003
FIN×Female	0.852	0.232	0.000
FRA×Female	0.570	0.161	0.000
GBR×Female	-0.414	0.196	0.034
GER×Female	0.858	0.160	0.000
HKG×Female	-0.442	0.839	0.599
HUN×Female	0.267	0.242	0.270
ITA×Female	0.364	0.167	0.029
JPN×Female	0.368	0.156	0.018
KOR×Female	0.074	0.468	0.875
NED×Female	0.613	0.243	0.011
POL×Female	1.219	0.241	0.000
RUS×Female	0.699	0.194	0.000
SUI×Female	0.950	0.173	0.000
SWE×Female	0.311	0.258	0.228
TPE×Female	-0.190	0.294	0.517
USA×Female	0.218	0.173	0.208

There is an omission of the R^2^ value in the legend of Table 13. There are two missing lines in Table 13 (race abroad and race abroad*female). Please see the correct Table 13 and correct legend below.

**Table 13:** Interaction with race site, results from truncated regression with complete data set time = sex×(year+year^2^)+sex×(age+age^2^) + sex×nationality×site and referenced to male, age 44, year 2009 and nationality Australia. R^2^ = 0.28.

	Coefficient	Standard error	P-Value
Intercept	14.043	0.0615	0.000
sex (female)	1.029	0.1296	0.000
age	0.013	0.0006	0.000
age squared	0.0032	0.0000	0.000
year	0.157	0.0013	0.000
year squared	0.0062	0.0000	0.000
age*female	0.021	0.0019	0.000
age squared*female	-0.0007	0.0001	0.000
year*female	0.009	0.0039	0.015
year squared*female	0.0016	0.0001	0.000
race abroad	-2.041	0.2219	0.000
race abroad*female	-0.8183	0.4246	0.054
AUS	0.000		
AUT	-3.621	0.1796	0.000
BEL	-3.480	0.1234	0.000
CAN	-1.300	0.1076	0.000
CHN	5.201	0.0869	0.000
CZE	2.448	0.1055	0.000
DEN	-3.661	0.1475	0.000
ESP	-0.399	0.0851	0.000
FIN	-3.773	0.1179	0.000
FRA	-1.332	0.0637	0.000
GBR	-2.477	0.1188	0.000
GER	-2.968	0.0673	0.000
HKG	4.780	0.1187	0.000
HUN	-3.101	0.1529	0.000
ITA	-0.511	0.0643	0.000
JPN	-2.961	0.0626	0.000
KOR	-1.707	0.0907	0.000
NED	-3.523	0.1151	0.000
POL	0.632	0.0801	0.000
RUS	-4.060	0.1229	0.000
SUI	-0.424	0.0656	0.000
SWE	-3.181	0.1519	0.000
TPE	-2.170	0.0910	0.000
USA	-0.012	0.0693	0.867
AUT*race abroad	3.994	0.2913	0.000
BEL*race abroad	4.684	0.2605	0.000
CAN*race abroad	2.645	0.2770	0.000
CHN*race abroad	1.420	0.2545	0.000
CZE*race abroad	-2.328	0.2665	0.000
DEN*race abroad	4.545	0.3366	0.000
ESP*race abroad	2.878	0.2568	0.000
FIN*race abroad	1.972	0.3570	0.000
FRA*race abroad	4.657	0.2278	0.000
GBR*race abroad	4.787	0.2561	0.000
GER*race abroad	3.308	0.2240	0.000
HKG*race abroad	-0.484	0.4050	0.232
HUN*race abroad	1.293	0.3396	0.000
ITA*race abroad	1.596	0.2406	0.000
JPN*race abroad	5.831	0.2636	0.000
KOR*race abroad	5.142	0.4134	0.000
NED*race abroad	4.362	0.2723	0.000
POL*race abroad	-0.624	0.2490	0.012
RUS*race abroad	0.267	0.2873	0.353
SUI*race abroad	2.078	0.2632	0.000
SWE*race abroad	3.296	0.3443	0.000
TPE*race abroad	2.539	0.3055	0.000
USA*race abroad	1.300	0.2495	0.000
AUT*female	-1.319	0.5884	0.025
BEL*female	-0.744	0.4024	0.064
CAN*female	0.792	0.2117	0.000
CHN*female	-0.483	0.2139	0.024
CZE*female	0.357	0.2593	0.169
DEN*female	-0.611	0.3860	0.113
ESP*female	0.849	0.2644	0.001
FIN*female	-0.351	0.2769	0.205
FRA*female	-0.068	0.1385	0.626
GBR*female	-0.251	0.2474	0.310
GER*female	-0.913	0.1526	0.000
HKG*female	0.650	0.2918	0.026
HUN*female	-1.180	0.4018	0.003
ITA*female	-0.491	0.1413	0.001
JPN*female	-0.781	0.1331	0.000
KOR*female	-0.311	0.3419	0.363
NED*female	-0.730	0.3210	0.023
POL*female	0.025	0.2143	0.906
RUS*female	-0.436	0.2716	0.109
SUI*female	0.442	0.1477	0.003
SWE*female	-1.042	0.3398	0.002
TPE*female	-0.522	0.3117	0.094
USA*female	0.092	0.1427	0.518
AUT* race abroad*female	1.282	0.7599	0.092
BEL* race abroad*female	1.295	0.6407	0.043
CAN* race abroad*female	-0.998	0.5163	0.053
CHN* race abroad*female	1.999	0.5403	0.000
CZE* race abroad*female	0.626	0.5920	0.291
DEN* race abroad*female	-0.217	0.7472	0.771
ESP* race abroad*female	2.805	0.6440	0.000
FIN* race abroad*female	3.390	0.7813	0.000
FRA* race abroad*female	0.269	0.4572	0.556
GBR* race abroad*female	-0.495	0.5127	0.335
GER* race abroad*female	1.253	0.4353	0.004
HKG* race abroad*female	0.018	0.8207	0.982
HUN* race abroad*female	1.499	0.6920	0.030
ITA* race abroad*female	-0.461	0.4999	0.356
JPN* race abroad*female	-1.423	0.4972	0.004
KOR* race abroad*female	2.612	1.0283	0.011
NED* race abroad*female	0.832	0.6342	0.189
POL* race abroad*female	2.097	0.5893	0.000
RUS* race abroad*female	1.199	0.5560	0.031
SUI* race abroad*female	-1.618	0.5741	0.005
SWE* race abroad*female	-0.180	0.7703	0.816
TPE* race abroad*female	-0.115	0.6995	0.870
USA* race abroad*female	-1.330	0.4787	0.005

There is an omission of the R^2^ value and an error in the legend of Table 14. There are two missing lines in Table 14 (race abroad and race abroad*female). Please see the correct Table 14 and correct legend below.

**Table 14:** Interaction with race site, results from linear regression with truncated dataset time = sex×(year+year^2^)+sex×(age+age^2^) + sex×nationality×site and referenced to male, age 44, year 2009, site at home and nationality Australia. R^2^ = 0.20.

	Coefficient	Standard error	P-Value
Intercept	11.319	0.0447	0.000
sex (female)	0.139	0.1045	0.182
age	0.020	0.0004	0.000
age squared	0.0011	0.0000	0.000
year	0.068	0.0008	0.000
year squared	0.0024	0.0000	0.000
age*female	0.005	0.0013	0.000
age squared*female	-0.0004	0.0001	0.000
year*female	0.006	0.0025	0.013
year squared*female	-0.0002	0.0001	0.152
race abroad	-1.747	0.1297	0.000
race abroad*female	0.1782	0.2556	0.486
AUS			
AUT	-1.092	0.0953	0.000
BEL	-1.228	0.0701	0.000
CAN	-0.128	0.0668	0.056
CHN	0.601	0.1196	0.000
CZE	-0.314	0.0984	0.001
DEN	-0.831	0.0792	0.000
ESP	-1.188	0.0601	0.000
FIN	-0.994	0.0667	0.000
FRA	-0.136	0.0458	0.003
GBR	-1.322	0.0737	0.000
GER	-1.040	0.0472	0.000
HKG	0.841	0.2060	0.000
HUN	-0.986	0.0867	0.000
ITA	0.336	0.0463	0.000
JPN	0.074	0.0451	0.102
KOR	0.432	0.0609	0.000
NED	-0.856	0.0658	0.000
POL	-0.105	0.0567	0.063
RUS	-1.672	0.0706	0.000
SUI	0.001	0.0469	0.986
SWE	-0.661	0.0835	0.000
TPE	0.510	0.0568	0.000
USA	0.273	0.0507	0.000
AUT*race abroad	2.484	0.1627	0.000
BEL*race abroad	2.343	0.1501	0.000
CAN*race abroad	0.957	0.1663	0.000
CHN*race abroad	1.571	0.2566	0.000
CZE*race abroad	1.002	0.1705	0.000
DEN*race abroad	1.025	0.1950	0.000
ESP*race abroad	1.297	0.1558	0.000
FIN*race abroad	0.860	0.1944	0.000
FRA*race abroad	1.484	0.1350	0.000
GBR*race abroad	2.418	0.1524	0.000
GER*race abroad	2.584	0.1308	0.000
HKG*race abroad	1.065	0.3543	0.003
HUN*race abroad	0.197	0.1876	0.295
ITA*race abroad	0.735	0.1427	0.000
JPN*race abroad	-0.399	0.1659	0.016
KOR*race abroad	0.012	0.2824	0.965
NED*race abroad	2.018	0.1583	0.000
POL*race abroad	0.864	0.1450	0.000
RUS*race abroad	-0.102	0.1583	0.520
SUI*race abroad	0.884	0.1622	0.000
SWE*race abroad	1.021	0.1956	0.000
TPE*race abroad	1.460	0.1714	0.000
USA*race abroad	0.567	0.1488	0.000
AUT*female	-0.564	0.3079	0.067
BEL*female	0.207	0.2213	0.349
CAN*female	0.324	0.1445	0.025
CHN*female	0.293	0.3774	0.437
CZE*female	-0.191	0.2767	0.489
DEN*female	0.408	0.2037	0.045
ESP*female	0.377	0.2155	0.081
FIN*female	0.567	0.1591	0.000
FRA*female	0.296	0.1089	0.007
GBR*female	-0.032	0.1626	0.845
GER*female	0.371	0.1127	0.001
HKG*female	0.798	0.8050	0.321
HUN*female	-0.139	0.2253	0.536
ITA*female	0.161	0.1113	0.149
JPN*female	0.144	0.1053	0.171
KOR*female	0.088	0.2539	0.729
NED*female	0.278	0.1813	0.125
POL*female	0.191	0.1639	0.244
RUS*female	0.284	0.1617	0.079
SUI*female	0.454	0.1155	0.000
SWE*female	0.024	0.1890	0.897
TPE*female	-0.121	0.1845	0.512
USA*female	0.234	0.1167	0.045
AUT* race abroad*female	0.658	0.4180	0.115
BEL* race abroad*female	-0.506	0.3768	0.179
CAN* race abroad*female	-0.052	0.3147	0.868
CHN* race abroad*female	-0.906	0.7296	0.214
CZE* race abroad*female	0.692	0.4175	0.098
DEN* race abroad*female	-0.199	0.4214	0.637
ESP* race abroad*female	1.056	0.5267	0.045
FIN* race abroad*female	0.958	0.4499	0.033
FRA* race abroad*female	-1.505	0.2853	0.000
GBR* race abroad*female	-1.157	0.3115	0.000
GER* race abroad*female	-0.119	0.2609	0.648
HKG* race abroad*female	-1.186	0.9940	0.233
HUN* race abroad*female	0.672	0.3816	0.078
ITA* race abroad*female	-1.355	0.3009	0.000
JPN* race abroad*female	-0.214	0.3048	0.482
KOR* race abroad*female	0.116	0.7603	0.878
NED* race abroad*female	-0.144	0.3698	0.697
POL* race abroad*female	0.900	0.3611	0.013
RUS* race abroad*female	0.585	0.3127	0.061
SUI* race abroad*female	-0.592	0.3508	0.091
SWE* race abroad*female	-0.452	0.4260	0.289
TPE* race abroad*female	-0.285	0.3870	0.461
USA* race abroad*female	-0.878	0.2887	0.002

In Table 16, column 3 was erroneously duplicated from column 4. Please see the correct Table 16 below.

**Table 16:** Comparing times in hours with finishes performed at home. Times were computed based on the model time = sex×(year+year^2^) +sex×(age+age^2^) + sex×nationality×site and referenced to male, age 44, year 2009 and nationality Australia.

	A		% Difference	B		% Difference	C		% Difference
	Data: complete		between	Data: truncated at 14 hours		between	Data: truncated at 14 hours		between
	Linear regression		Races abroad	Linear regression		races at home	Truncated linear regression		Races abroad
	with fixed effects		/at home	with fixed effects		/on abroad	with fixed effects		/at home
	Races at home	Races abroad		Races at home	Races abroad		Races at home	Races abroad	
AUS	14.0	12.0	-14.5%	11.3	9.6	-15.4%	11.7	9.6	-17.8%
AUT	10.4	12.4	18.7%	10.2	11.0	7.2%	10.5	11.4	9.3%
BEL	10.6	13.2	25.0%	10.1	10.7	5.9%	10.2	10.9	6.5%
CAN	12.7	13.3	4.7%	11.2	10.4	-7.1%	11.7	10.6	-9.4%
CHN	19.2	18.6	-3.2%	11.9	11.7	-1.5%	13.1	13.2	0.7%
CZE	16.5	12.1	-26.5%	11.0	10.3	-6.8%	11.3	10.3	-8.8%
DEN	10.4	12.9	24.1%	10.5	9.8	-6.9%	10.7	9.9	-7.2%
ESP	13.6	14.5	6.1%	10.1	9.7	-4.4%	10.3	10.0	-3.2%
FIN	10.3	10.2	-0.7%	10.3	9.4	-8.6%	10.4	9.3	-10.6%
FRA	12.7	15.3	20.6%	11.2	10.9	-2.4%	11.6	11.2	-3.4%
GBR	11.6	14.3	23.7%	10.0	10.7	6.7%	10.1	10.9	8.1%
GER	11.1	12.3	11.4%	10.3	11.1	8.1%	10.4	11.5	10.6%
HKG	18.8	16.3	-13.4%	12.2	11.5	-5.6%	13.1	11.2	-14.5%
HUN	10.9	10.2	-6.8%	10.3	8.8	-15.0%	10.5	8.9	-15.3%
ITA	13.5	13.1	-3.3%	11.7	10.6	-8.7%	12.4	10.8	-12.9%
JPN	11.1	14.9	34.2%	11.4	9.2	-18.8%	12.0	9.4	-21.4%
KOR	12.3	15.4	25.1%	11.8	10.0	-14.8%	12.8	10.3	-19.4%
NED	10.5	12.8	22.1%	10.5	10.7	2.6%	10.6	11.0	4.0%
POL	14.7	12.0	-18.2%	11.2	10.3	-7.9%	11.6	10.5	-9.2%
RUS	10.0	8.2	-17.8%	9.6	7.8	-19.2%	9.5	7.9	-17.2%
SUI	13.6	13.7	0.3%	11.3	10.5	-7.6%	11.8	10.4	-12.3%
SWE	10.9	12.1	11.6%	10.7	9.9	-6.8%	10.7	9.8	-8.5%
TPE	11.9	12.4	4.2%	11.8	11.5	-2.4%	12.9	12.2	-5.8%
USA	14.0	13.3	-5.3%	11.6	10.4	-10.2%	12.4	10.6	-14.1%

The authors provide the following additional information:

After the publication of this article [1], concerns were raised regarding the data analysis presented in [1]. The dataset from 1959 to 2000 is limited and an additional data analysis including only races held since 2000 was performed. For the regression, the median centered age and running year was used. Median of age changed from 44 to 46 and year changed from 2009 to 2012. The number of finishes decreased 32% (from 307,871 to 209,776) by removing finishes before 2000, however, the analysis remains robust. This can be shown by comparing the originally published Fig 9 with the new analysis presented in S1 Fig below.

There are minor changes in the ranks but the main conclusions about Russia and Japan remain the same.

All other results (estimates from regression, distributions, etc.) are only slightly affected. For example, comparing the time estimates in Fig 9 (A to C) and S1 Fig (A to C), there is a shift towards higher running time in each regression type. This can be explained by increased participation of amateur after 1990. But this does not affect the ranks.

Additionally, the distributions do not change dramatically. The main groupings (1 = CHN,HKG), (2 = RUS) and (3 = KOR,JAP,TPE) remain, whereas groups (4) and (5) changed slightly: France, Canada and Poland changed from group 5 to group 4 which has a slight lower skewness than group 5. These changes should not be overinterpreted since these both groups are very similar. This can be seen comparing the new analysis in S2 Fig and S3 Fig with the originally published Fig 3 and Fig 4, respectively. In summary, the new analysis confirms the conclusion in the original analysis.

The authors provide the following updated information regarding the regression analysis:

Linear regression with truncated data gives biased estimates. The bias can be omitted or attenuated using truncated regression. To see if the conclusion from linear regression can be confirmed we used a method (trunc reg), which considers the bias due to truncated data. To compare finishes with similar conditions as in Japan, which have a cut at about 15 hours, we decided to truncate the data at 15 hours in all nations.

## Supporting information

S1 Fig The upper panel shows the adjusted time for each nationality in ascending order at reference sex = male, age = 46 and year = 2012 including only finishes since 2000. (A) is based on linear regression of the complete dataset, (B) on the truncated dataset and (C) on the truncated regression of the truncated dataset. The lower panel with figures (D) and (E) shows the changes in rank from (A) to (B) and (B) to (C).
(TIF)

S2 Fig Histograms, density plots and normal distributions based on mean and standard deviation for each country including only finishes since 2000. The diagrams are positioned according to the hierarchical cluster analysis. Graphs are based on the complete dataset.
(TIF)

S3 Fig Scatterplot with excess against skewness including only finishes since 2000. Groups of nation are distinguished by different colours.
(TIF)
