# Supplementary figures and images for: Correction: Russians are the fastest 100-km ultra-marathoners in the world
Source: PLoS One. 2022 Jul 21;17(7):e0272170. doi: 10.1371/journal.pone.0272170 (PMC9302838; doi:10.1371/journal.pone.0272170)

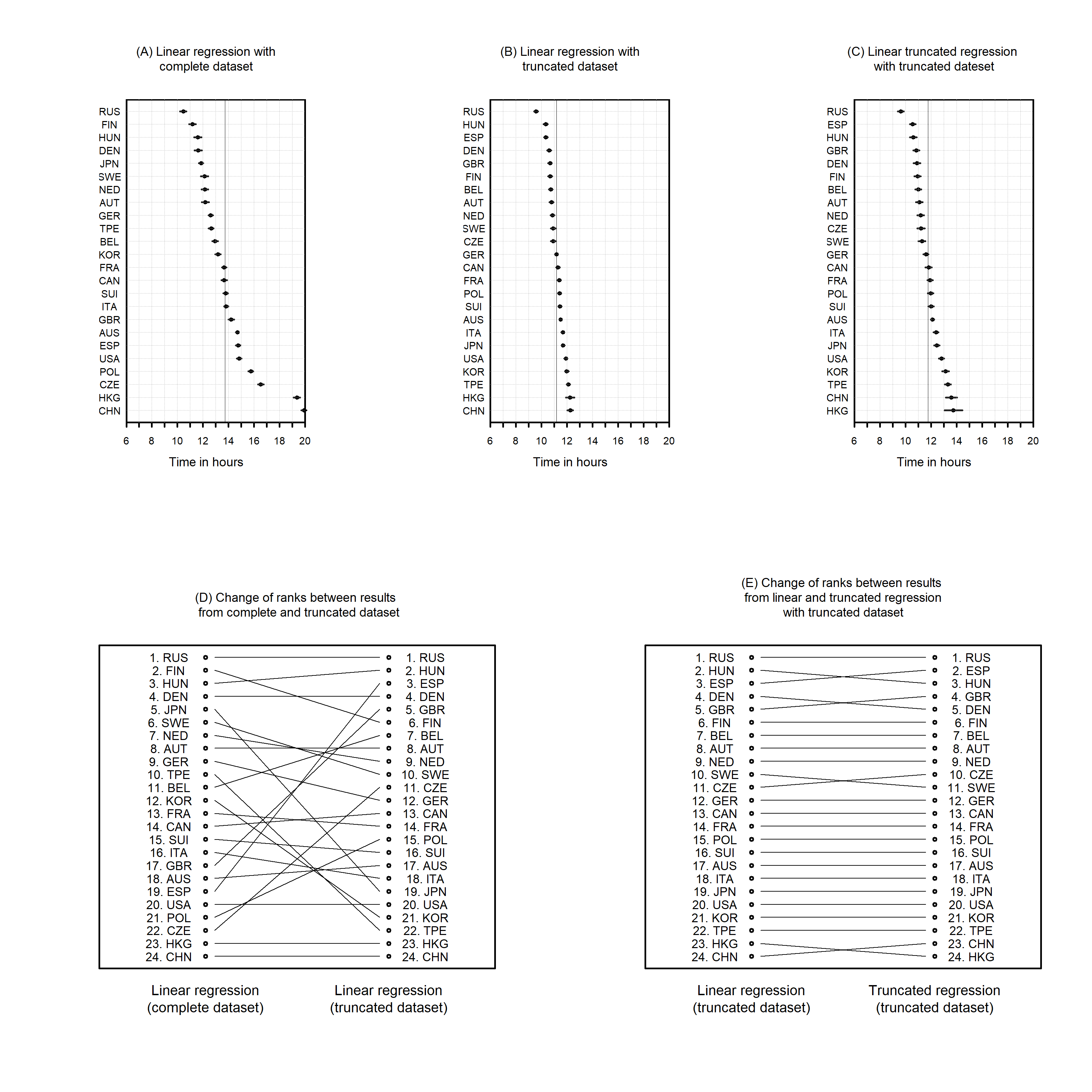

Supplement: S1 Fig — (A) is based on linear regression of the complete dataset, (B) on the truncated dataset and (C) on the truncated regression of the truncated dataset. The lower panel with figures (D) and (E) shows the changes in rank from (A) to (B) and (B) to (C). (TIF) [file pone.0272170.s001.tif]

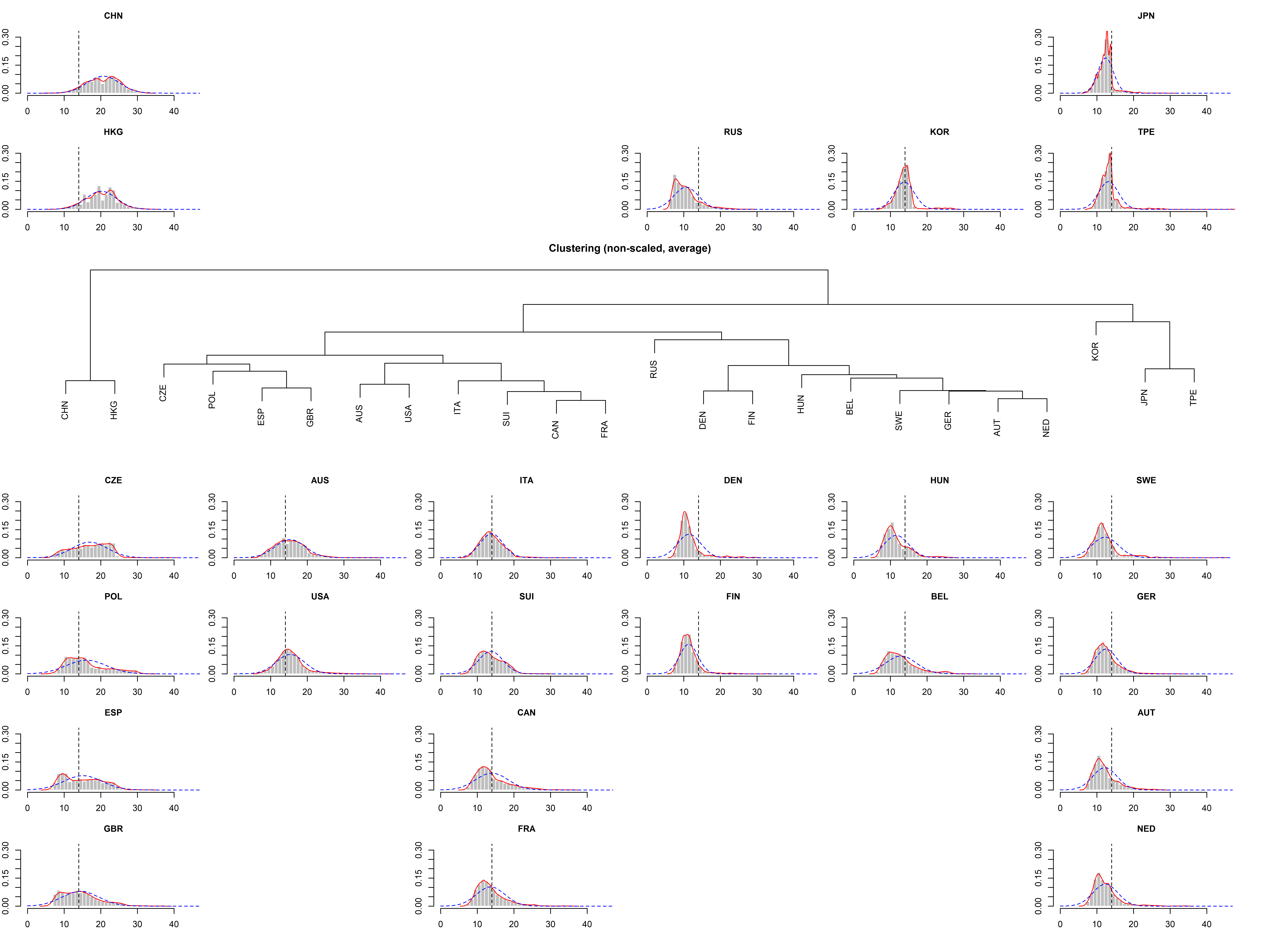

Supplement: S2 Fig — The diagrams are positioned according to the hierarchical cluster analysis. Graphs are based on the complete dataset. (TIF) [file pone.0272170.s002.tif]

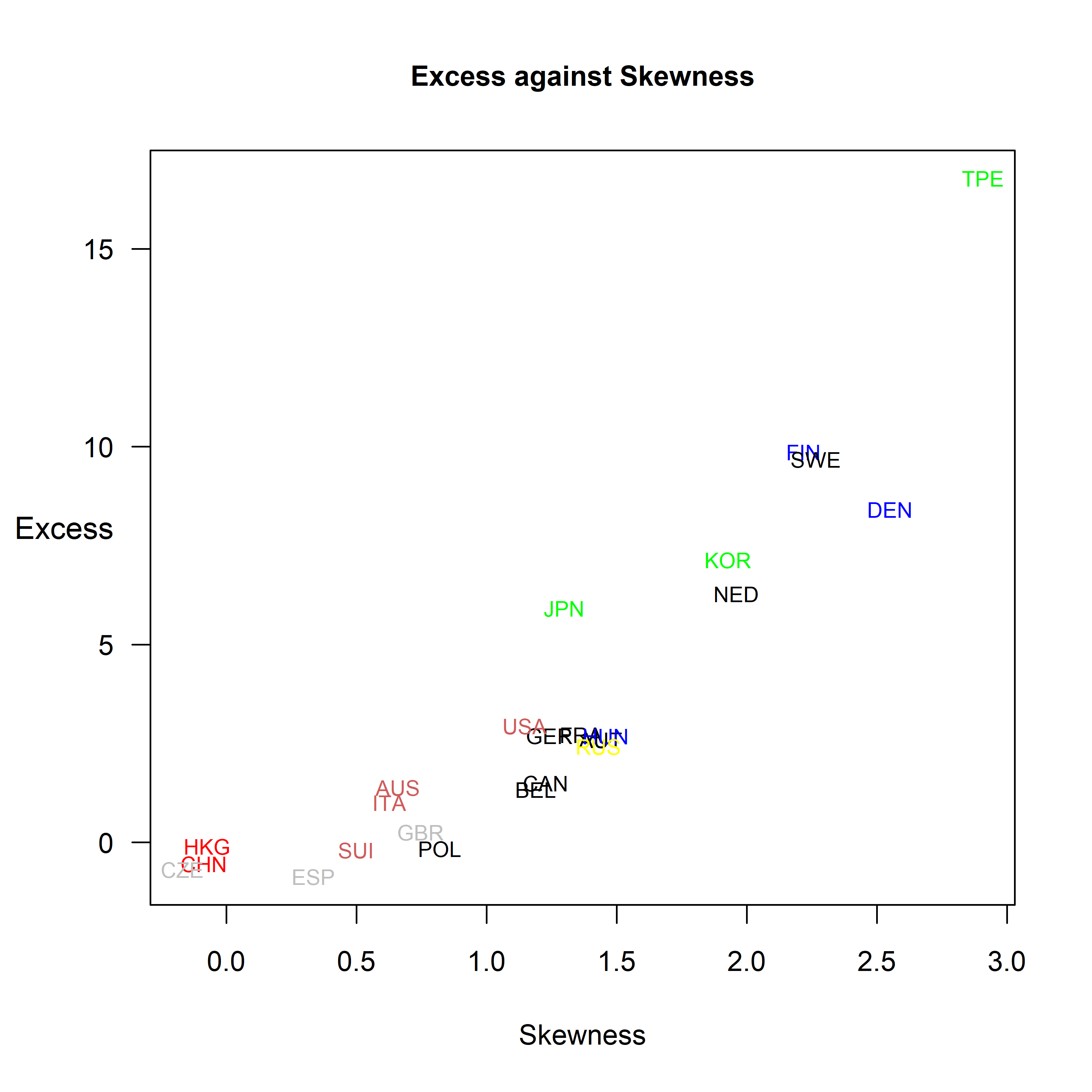

Supplement: S3 Fig — Groups of nation are distinguished by different colours. (TIF) [file pone.0272170.s003.tif]
